# Supplementary material for: Unveiling the Potential of BenzylethyleneAryl–Urea Scaffolds for the Design of New Onco Immunomodulating Agents
Source: Pharmaceuticals (Basel). 2023 May 29;16(6):808. doi: 10.3390/ph16060808 (PMC10302582; doi:10.3390/ph16060808)
Supplement: Supplementary file 1 [file pharmaceuticals-16-00808-s001.zip › pharmaceuticals-2390615-supplementary.pdf]

Unveiling the Potential of BenzylethyleneAryl-Urea Scaffolds for Designing of new Onco Immunomodulating Agents

Raquel Gil-Edo [1], Santiago Royo [1], Miguel Carda [1,], Eva Falomir [1,*]

[1] Depart. de Q. Inorgánica y Orgánica, Univ. Jaume I, E-12071 Castellón, Spain.
* Correspondence: efalomir@uji.es

Supporting Information

Contents:

Experimental procedure .... **- 1 -**

Analytical NMR spectra .... **- 3 -**

Graphical NMR spectra .... **- 13 -**

Experimental procedure

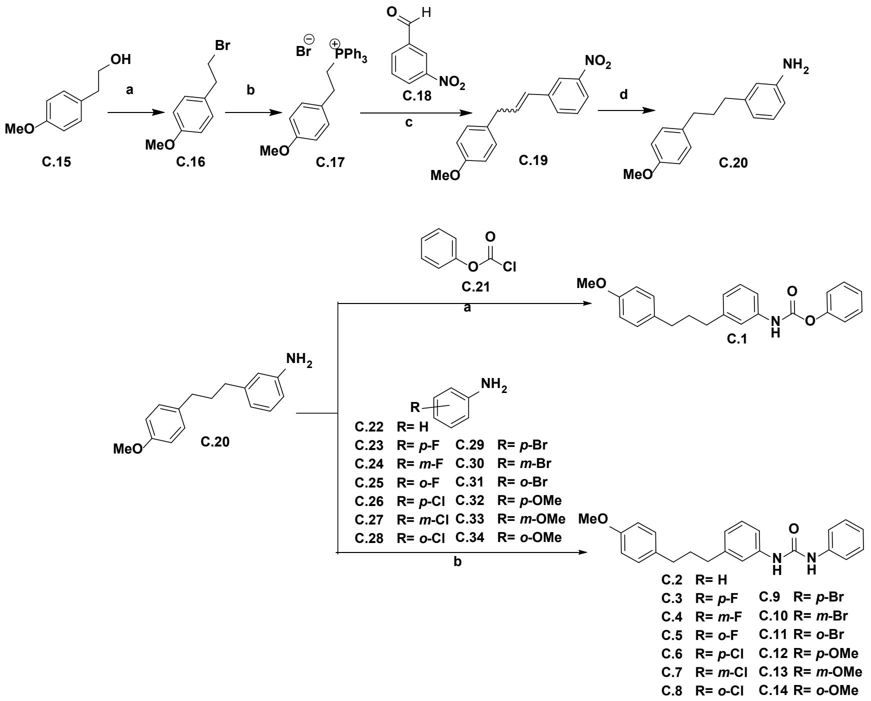

1-(Bromomethyl)-4-methoxybenzene (C.16)

To a solution of 2-(4-methoxyphenyl)ethan-1-ol **C.15** (3.88 g, 25.3 mmol) in toluene (33 mL) was added PBr_3 (856 µL, 8.8 mmol, 0.35 eq) under nitrogen atmosphere and the resulting mixture was refluxed for 2 hr. After cooling to room temperature the mixture was washed with and aqueous saturated solution of $Na_2S_2O_3/NaHCO_3$ (1:1) (3 x 20 mL), dried over $MgSO_4$ and concentrated *in vacuo* to give 5.12 g (94 %)of compound **C.16** as a clear oil.

(4-Methoxyphenethyl)triphenylphosphonium bromide (C.17)

To a solution of 1-(2-bromoethyl)-4-methoxybenzene **C.16** (5.12 g, 23.80 mmol) in EtOH (240 mL) was added PPh_3 (7.00 g, 26.20 mmol, 1.1 eq). The mixture was refluxed for 6 days under nitrogen atmosphere. Then, the solvent was removed *in vacuo* and the crude product was purified on silica gel chromatography using dichloromethane and dichloromethane:MeOH (9:1) to give 9.44 g (83%) of **C.17** as a clear oil.

(*E/Z*)-1-(3-(4-Methoxyphenyl)prop-1-en-1-yl)-3-nitrobenzene (C.19)

To a solution of (4-methoxyphenethyl)triphenylphosphonium bromide **C.17** (3.11 g, 6.51 mmol) in dichloromethane (40 mL) was added 3-nitrobenzaldehyde **C.18** (994 mg, 6.51 mmol), K_2CO_3 (7.16 mmol, 1.1 eq) and 18-crown-6 (310 mg, 0.18 eq). The mixture was refluxed for 24 h and then filtered and concentrated *in vacuo*. The crude product was purified on silica gel chromatography using Hexanes:AcOEt (9:1) to give 1.38 g (79 %) of compound **C.19** as an orange oil.

3-(3-(4-Methoxyphenyl)propyl)aniline (C.20)

A solution of (*E/Z*)-1-(3-(4-methoxyphenyl)prop-1-en-1-yl)-3-nitrobenzene **C.19** (860 mg, 3.19 mmol) in AcOEt (19 mL) was added to a mixture of Pd/C (10%, 172 mg) in AcOEt (3.5 mL). The resulting mixture was stirred overnight under H_2 atmosphere and then filtered through Celite yielding 602 mg (78 %) of compound **C.19** as a brown oil.

(Phenyl (3-((4-methoxybenzyl)oxy)phenyl)carbamate) (C.1)

A solution of 3-(3-(4-methoxyphenyl)propyl)aniline **C.20** (361 mg, 1.5 mmol) in dry THF (7.5 mL) was cooled at 0 ºC and then anhydrous pyridine (301 µL, 3.74 mmol) and phenyl chloroformiate **C.21** (291 µL, 2.25 mmol) were added. The resulting mixture was stirred under nitrogen atmosphere for 20 min at 0ºC and at room temperature for 1 h. Then, water (7.5 mL) and 1M aqueous HCl (3.75 mL) were added and the mixture was extracted with CH_2Cl_2 (3 x 20 mL). The combined organic phases were washed with brine and dried over Na_2SO_4. The solvent was removed *in vacuo* and the residue was purified on silica gel chromatography using Hexanes:Ethyl acetate (9:1) as eluent yielding 384 mg (71 %) of carbamate **C.1** as a white solid.

Analytical NMR spectra

1-(2-Bromoethyl)-4-methoxybenzene (C.16). Yield 94%; clear oil; ^{1}H NMR (400 MHz, $CDCl_3$); δ 7.15 (d, J = 8.7 Hz, 2H), 6.89 (d, J = 8.7 Hz, 2H), 3.82 (s, 3H), 3.55 (t, J = 7.6 Hz, 2H), 3.13 (t, J = 7.6 Hz, 2H).; ^{13}C NMR (100 MHz, $CDCl_3$) δ 158.52 (C), 130.97 (C), 129.59 (2CH), 113.97 (2CH), 55.18 (CH3), 38.53 (CH2), 33.29 (CH2).

(4-Methoxyphenethyl)triphenylphosphonium bromide (C.17). Yield 94%; clear oil; ^{1}H NMR (400 MHz, $CDCl_3$) δ 7.89 – 7.76 (m, 9H), 7.73 – 7.66 (m, 6H), 7.22 (d, J = 8.7 Hz, 2H), 6.77 (d, J = 8.7 Hz, 2H), 4.16 – 4.05 (m, 2H), 3.75 (s, 3H), 3.07 – 2.95 (m, 2H).; ^{13}C NMR (100 MHz, $CDCl_3$) δ 158.65 (C), 135.00 (3CH, d, J = 3.0 Hz), 133.77 (6CH, d, J = 10.1 Hz), 130.47 (6CH, d, J = 12.6 Hz), 130.14 (C, d, J = 13.6 Hz), 129.70 (2CH), 118.22 (3C, d, J = 85.7 Hz), 114.27 (2CH), 55.29 (CH3), 27.61 (CH2, d, J = 3.7 Hz), 24.87 (CH2, d, J = 47.4 Hz).

(*Z*)-1-(3-(4-Methoxyphenyl)prop-1-en-1-yl)-3-nitrobenzene + (*E*)-1-(3-(4-methoxyphenyl)prop-1-en-1-yl)-3-nitrobenzene (42:58) (C.19). Yield 91%; orange oil; ^{1}H NMR (400 MHz, $CDCl_3$) δ 8.23 – 8.16 (m, 2H), 8.12 (ddd, J = 8.2, 2.2, 1.0 Hz, 1H-cis), 8.05 (ddd, J = 8.2, 2.3, 1.0 Hz, 1H-trans), 7.64 (ddd, J = 7.7, 3.3, 2.1 Hz, 2H), 7.52 (t, J = 7.9 Hz, 1H-cis), 7.45 (t, J = 8.0 Hz, 1H-trans), 7.17 (d, J = 8.7 Hz, 2H-trans), 7.14 (d, J = 8.8 Hz, 2H-cis), 6.89 (d, J = 8.7 Hz, 2H-trans), 6.87 (d, J = 8.6 Hz, 2H-cis), 6.60 (d, J = 11.5 Hz, 1H-cis), 6.51 (dd, J = 15.3, 9.2 Hz, 1H-trans), 6.46 (d, J = 15.9 Hz, 1H-trans), 6.04 (dt, J = 11.5, 7.6 Hz, 1H-cis), 3.82 (s, 3H-trans), 3.81 (s, 3H-cis), 3.61 (dd, J = 7.6, 1.6 Hz, 2H-cis), 3.54 (d, J = 5.7 Hz, 2H-trans).; ^{13}C NMR (100 MHz, $CDCl_3$) δ 158.29 (C), 158.20 (C), 148.60 (C), 148.29 (C), 139.32 (C), 138.82 (C), 134.57 (CH), 134.02 (CH), 133.29 (CH), 131.88 (CH), 131.78 (C), 131.20 (C), 129.63 (2CH), 129.31 (CH), 129.17 (CH), 129.13 (2CH), 128.49 (CH), 127.40 (CH), 123.45 (CH), 121.62 (CH), 121.60 (CH), 120.67 (CH), 114.10 (2CH), 114.07 (2CH), 55.28 (CH3), 55.26 (CH3), 38.34 (CH2), 33.65 (CH2); HR ESMS *m/z* 292.0956 [M-Na]+. Calc. for $C_{16}H_{15}NO_3$ 292.0950.

3-(3-(4-Methoxyphenyl)propyl)aniline (C.20). Yield 78%; brown oil; ^{1}H NMR (400 MHz, $CDCl_3$) δ 7.12 (d, J = 8.8 Hz, 2H), 7.10 – 7.05 (m, 1H), 6.85 (d, J = 8.7 Hz, 2H), 6.62 (d, J = 7.8 Hz, 1H), 6.57 – 6.52 (m, 2H), 3.81 (s, 3H), 3.52 (s, 2H), 2.64 – 2.52 (m,

4H), 1.98 – 1.85 (m, 2H).; ^{13}C NMR (100 MHz, $CDCl_3$) δ 157.64 (C), 146.30 (C), 143.56 (C), 134.40 (C), 129.23 (2CH), 129.09 (CH), 118.77 (CH), 115.22 (CH), 113.66 (2CH), 112.57 (CH), 55.16 (CH3), 35.30 (CH2), 34.46 (CH2), 32.93 (CH2); HR ESMS *m/z* 242.1540 [M-H]+. Calc. for $C_{16}H_{19}NO$ 242.1545.

Phenyl (3-(3-(4-methoxyphenyl)propyl)phenyl)carbamate (C.1). Yield 71%; white solid; m.p. 89-91ºC; ^{1}H NMR (400 MHz, $CDCl_3$); δ 7.37 – 7.29 (m, 2H), 7.26 (s, 1H), 7.22 – 7.10 (m, 5H), 7.03 (d, J = 8.7 Hz, 2H), 6.96 – 6.83 (m, 2H), 6.77 (d, J = 8.7 Hz, 2H), 3.72 (s, 3H), 2.61 – 2.48 (m, 4H), 1.86 (tt, J = 9.1, 6.9 Hz, 2H).; ^{13}C NMR (100 MHz, $CDCl_3$) δ 157.69 (C), 151.63 (C), 150.56 (C), 143.60 (C), 137.31 (C), 134.21 (C), 129.35 (2CH), 129.27 (2CH), 128.96 (CH), 125.63 (CH), 124.08 (CH), 121.62 (2CH), 118.78 (CH), 116.18 (CH), 113.72 (2CH), 55.21 (CH3), 35.32 (CH2), 34.44 (CH2), 33.00 (CH2).; IR $_{vmax}$ (cm-1) 3296 (N-H), 1706 (C=O), 1545 (N-H amide), 1202,1172,1028 (C-O).; HR ESMS *m/z* 384.1579 [M-Na]+. Calc. for $C_{23}H_{23}NO_3$ 384.1576.

1-(3-(3-(4-Methoxyphenyl)propyl)phenyl)-3-phenylurea (C.2). Yield 52%; white solid; m.p. 157-159ºC; ^{1}H NMR (400 MHz,DMSO); δ 8.60 (s, 1H), 8.57 (s, 1H), 7.45 (dd, J = 8.6, 1.1 Hz, 2H), 7.32 (d, J = 1.7 Hz, 1H), 7.30 – 7.21 (m, 3H), 7.17 (t, J = 7.7 Hz, 1H), 7.12 (d, J = 8.7 Hz, 2H), 7.00 – 6.92 (m, 1H), 6.84 (d, J = 8.7 Hz, 2H), 6.80 (d, J = 7.4 Hz, 1H), 3.72 (s, 3H), 2.54 (dd, J = 13.4, 5.7 Hz, 4H), 1.84 (dq, J = 15.3, 7.7 Hz, 2H).; ^{13}C NMR (100 MHz, DMSO) δ 157.34 (C), 152.48 (C), 142.55 (C), 139.68 (C), 139.62 (C), 133.74 (C), 129.14 (2CH), 128.71 (2CH), 128.61 (CH), 121.85 (CH), 121.72 (CH), 118.14 (2CH), 118.02 (CH), 115.68 (CH), 113.67 (2CH), 54.91 (CH3), 34.74 (CH2), 33.77 (CH2), 32.82 (CH2).; IR $_{vmax}$ (cm-1) 3299 (N-H), 1643 (C=O), 1554 (N-H amide), 1446 (N-C=O), 1242,1041 (C-O).; HR ESMS *m/z* 383.1728 [M-Na]+. Calc. for $C_{23}H_{24}N_2O_2$ 383.1736.

1-(4-Fluorophenyl)-3-(3-(3-(4-methoxyphenyl)propyl)phenyl)urea (C.3). Yield 91%; white solid; m.p. 181-183ºC; ^{1}H NMR (400 MHz,DMSO); δ 8.63 (s, 1H), 8.56 (s, 1H), 7.45 (dd, J = 9.2, 4.9 Hz, 2H), 7.31 (t, J = 1.7 Hz, 1H), 7.25 – 7.20 (m, 1H), 7.17 (t, J = 7.7 Hz, 1H), 7.14 – 7.05 (m, 4H), 6.87 – 6.82 (m, 2H), 6.80 (d, J = 7.4 Hz, 1H), 3.72 (s, 3H), 2.57 – 2.52 (m, 4H), 1.89 – 1.76 (m, 2H).; ^{13}C NMR (100 MHz, DMSO) δ 157.86 (s), 157.80 (C, J = 238.0 Hz), 153.10 (C), 143.06 (C), 140.11 (C), 136.55 (C), 134.26 (C), 129.66 (2CH), 129.12 (CH), 122.40 (CH), 120.43 (2CH, d, J = 7.7 Hz), 118.60 (CH),

116.26 (CH), 115.71 (2CH, d, J = 22.2 Hz), 114.19 (2CH), 55.43 (CH3), 35.25 (CH2), 34.29 (CH2), 33.33 (CH2).; 19F NMR (377 MHz, DMSO) δ -121.58; IR $_{vmax}$ (cm-1) 3292 (N-H), 1637 (C=O), 1561 (N-H amide), 1508 (N-C=O), 1212, 1041 (C-O).; HR ESMS *m/z* 401.1636 [M-Na]+. Calc. for $C_{23}H_{23}N_2O_2F$ 401.1642.

1-(3-Fluorophenyl)-3-(3-(3-(4-methoxyphenyl)propyl)phenyl)urea (C.4). Yield 88%; white solid; m.p. 154ºC; ^{1}H NMR (400 MHz,DMSO); δ 8.87 (s, 1H), 8.67 (s, 1H), 7.49 (dt, J = 12.1, 2.3 Hz, 1H), 7.32 (t, J = 2.0 Hz, 1H), 7.31 – 7.26 (m, 1H), 7.23 (tt, J = 4.5, 2.2 Hz, 1H), 7.18 (t, J = 7.7 Hz, 1H), 7.12 (d, J = 8.6 Hz, 2H), 7.11 – 7.07 (m, 1H), 6.84 (d, J = 8.7 Hz, 2H), 6.83 – 6.80 (m, 1H), 6.80 – 6.72 (m, 1H), 3.72 (s, 3H), 2.55 (t, J = 7.6 Hz, 4H), 1.90 – 1.77 (m, 2H).; ^{13}C NMR (100 MHz, DMSO) δ 162.37 (C, J = 240.7 Hz), 157.35 (C), 152.34 (C), 142.59 (C), 141.60 (C, d, J = 11.4 Hz), 139.34 (C), 133.74 (C), 130.22 (CH, d, J = 9.8 Hz), 129.15 (2CH), 128.64 (CH), 122.11 (CH), 118.20 (CH), 115.87 (CH), 113.85 (CH, d, J = 2.6 Hz), 113.67 (2CH), 108.01 (CH, d, J = 21.4 Hz), 104.79 (CH, d, J = 26.5 Hz), 54.91 (CH3), 34.72 (CH2), 33.77 (CH2), 32.81 (CH2).; 19F NMR (377 MHz, DMSO) δ -112.31; IR $_{vmax}$ (cm-1) 3292 (N-H), 1640 (C=O), 1554 (N-H amide), 1508 (N-C=O), 1245, 1143, 1035 (C-O).; HR ESMS *m/z* 401.1640 [M-Na]+. Calc. for $C_{23}H_{23}N_2O_2F$ 401.1642.

1-(2-Fluorophenyl)-3-(3-(3-(4-methoxyphenyl)propyl)phenyl)urea (C.5). Yield 92%; white solid; m.p. 152ºC; ^{1}H NMR (400 MHz,DMSO); δ 9.00 (s, 1H), 8.49 (d, J = 2.5 Hz, 1H), 8.15 (td, J = 8.3, 1.7 Hz, 1H), 7.32 (t, J = 1.7 Hz, 1H), 7.27 – 7.21 (m, 2H), 7.20 – 7.14 (m, 2H), 7.12 (d, J = 8.6 Hz, 2H), 7.04 – 6.95 (m, 1H), 6.84 (d, J = 8.7 Hz, 2H), 6.82 (m, 1H), 3.72 (s, 3H), 2.60 – 2.52 (m, 4H), 1.90 – 1.76 (m, 2H).; ^{13}C NMR (100 MHz, DMSO)) δ 157.35 (C), 152.14 (C), 151.96 (C, d, J = 241.1 Hz), 142.68 (C), 139.36 (C), 133.75 (C), 129.15 (2CH), 128.72 (CH), 127.54 (d, J = 10.3 Hz), 124.44 (s), 122.35 (d, J = 7.5 Hz), 122.12 (s), 120.54 (C), 117.94 (CH), 115.61 (CH), 114.89 (CH, d, J = 19.1 Hz), 113.68 (2CH), 54.92 (CH3), 34.73 (CH2), 33.77 (CH2), 32.81 (CH2).; 19F NMR (377 MHz, DMSO) δ -129.80; IR $_{vmax}$ (cm-1) 3302 (N-H), 1647 (C=O), 1554 (N-H amide), 1508 (N-C=O), 1238, 1196, 1047 (C-O).; HR ESMS *m/z* 401.1639 [M-Na]+. Calc. for $C_{23}H_{23}N_2O_2F$ 401.1641.

1-(4-Chlorophenyl)-3-(3-(3-(4-methoxyphenyl)propyl)phenyl)urea (C.6). Yield 66%; white solid; m.p. 179-182ºC; ^{1}H NMR (400 MHz,DMSO); δ 8.75 (s, 1H), 8.61 (s, 1H), 7.48 (d, J = 9.0 Hz, 2H), 7.34 – 7.28 (m, 3H), 7.25 – 7.21 (m, 1H), 7.18 (t, J = 7.7 Hz, 1H), 7.12 (d, J = 8.7 Hz, 2H), 6.84 (d, J = 8.7 Hz, 2H), 6.83 – 6.79 (m, 1H), 3.72 (s, 3H), 2.55 (t, J = 7.7 Hz, 4H), 1.89 – 1.78 (m, 2H).; ^{13}C NMR (100 MHz, DMSO) δ 157.34(C), 152.38 (C), 142.57(C), 139.43(C), 138.70 (C), 133.74 (C), 129.14 (2CH), 128.62 (C), 128.54 (2CH), 122.54(CH), 122.03 (CH), 119.67 (2CH), 118.15 (CH), 115.81 (CH), 113.67 (2CH), 54.91 (CH3), 34.71 (CH2), 33.76 (CH2), 32.80 (CH2).; IR $_{vmax}$ (cm-1) 3289 (N-H), 1637 (C=O), 1558 (N-H amide), 1505 (N-C=O), 1244, 1044 (C-O).; HR ESMS *m/z* 417.1339 [M-Na]+. Calc. for $C_{23}H_{23}N_2O_2Cl$ 417.1346.

1-(3-Chlorophenyl)-3-(3-(3-(4-methoxyphenyl)propyl)phenyl)urea (C.7). Yield 69%; white solid; m.p. 143-147ºC; ^{1}H NMR (400 MHz,DMSO); δ 8.83 (s, 1H), 8.66 (s, 1H), 7.72 (t, J = 1.9 Hz, 1H), 7.33 (t, J = 1.7 Hz, 1H), 7.32 – 7.27 (m, 1H), 7.27 – 7.22 (m, 2H), 7.22 – 7.16 (m, 1H), 7.13 (d, J = 8.7 Hz, 2H), 7.01 (ddd, J = 7.6, 2.1, 1.4 Hz, 1H), 6.85 (d, J = 8.7 Hz, 2H), 6.84 – 6.79 (m, 1H), 3.73 (s, 3H), 2.55 (t, J = 7.6 Hz, 4H), 1.91 – 1.77 (m, 2H).; ^{13}C NMR (100 MHz, DMSO) δ 157.87 (C), 152.85 (C), 143.11 (C), 141.80 (C), 139.85 (C), 134.25 (C), 133.67 (C), 130.83 (CH), 129.67 (2CH), 129.15 (CH), 122.65 (CH), 121.85 (CH), 118.75 (CH), 118.01 (CH), 117.09 (CH), 116.41 (CH), 114.19 (2CH), 55.43 (CH3), 35.24 (CH2), 34.29 (CH2), 33.33 (CH2).; IR $_{vmax}$ (cm-1) 3299 (N-H), 1637 (C=O), 1547 (N-H amide), 1511 (N-C=O), 1244, 1035 (C-O).; HR ESMS *m/z* 417.1341 [M-Na]+. Calc. for $C_{23}H_{23}N_2O_2Cl$ 417.1346.

1-(2-Chlorophenyl)-3-(3-(3-(4-methoxyphenyl)propyl)phenyl)urea (C.8). Yield 92%; white solid; m.p. 134-136ºC; ^{1}H NMR (400 MHz,DMSO); δ 9.34 (s, 1H), 8.26 (s, 1H), 8.16 (dd, J = 8.3, 1.5 Hz, 1H), 7.45 (dd, J = 8.0, 1.4 Hz, 1H), 7.33 (t, J = 1.7 Hz, 1H), 7.32 – 7.27 (m, 1H), 7.27 – 7.23 (m, 1H), 7.20 (t, J = 7.7 Hz, 1H), 7.12 (d, J = 8.7 Hz, 2H), 7.02 (ddd, J = 8.0, 7.4, 1.6 Hz, 1H), 6.85 (d, J = 8.2 Hz, 2H), 6.83 – 6.81 (m, 1H), 3.72 (s, 3H), 2.55 (td, J = 7.8, 4.0 Hz, 4H), 1.91 – 1.76 (m, 2H).; ^{13}C NMR (100 MHz, DMSO) δ 157.35 (C), 152.07 (C), 142.66 (C), 139.37 (C), 135.96 (C), 133.73 (C), 129.14 (3CH), 128.73 (CH), 127.50 (CH), 123.19 (CH), 122.18 (CH), 121.83 (C), 121.26 (CH), 118.02 (CH), 115.68 (CH), 113.68 (2CH), 54.91 (CH3), 34.72 (CH2), 33.76 (CH2), 32.80 (CH2).; IR $_{vmax}$ (cm-1) 3266 (N-H), 1647 (C=O), 1561 (N-H amide), 1508 (N-C=O),

1249, 1041 (C-O).; HR ESMS *m/z* 417.1340 [M-Na]+. Calc. for $C_{23}H_{23}N_2O_2Cl$ 417.1346.

1-(4-Bromophenyl)-3-(3-(3-(4-methoxyphenyl)propyl)phenyl)urea (C.9.). Yield 51%; white solid; m.p. 191-196ºC; ^{1}H NMR (400 MHz,DMSO); δ 8.75 (s, 1H), 8.61 (s, 1H), 7.43 (s, 4H), 7.31 (d, J = 1.6 Hz, 1H), 7.25 – 7.20 (m, 1H), 7.18 (t, J = 7.7 Hz, 1H), 7.12 (d, J = 8.7 Hz, 2H), 6.84 (d, J = 8.7 Hz, 2H), 6.81 (d, J = 7.5 Hz, 1H), 3.72 (s, 3H), 2.54 (dd, J = 13.0, 5.2 Hz, 4H), 1.91 – 1.76 (m, 2H).; ^{13}C NMR (100 MHz, DMSO) δ 156.29 (C), 151.29 (C), 141.52 (C), 138.36 (C), 138.09 (C), 132.69 (C), 130.39 (2CH), 128.09 (2CH), 127.58 (CH), 121.00 (CH), 119.03 (2CH), 117.11 (CH), 114.77 (CH), 112.62 (2CH), 112.04 (C), 53.86 (CH3), 33.67 (CH2), 32.72 (CH2), 31.75 (CH2).; IR $_{vmax}$ (cm-1) 3296 (N-H), 1640 (C=O), 1548 (N-H amide), 1508 (N-C=O), 1244, 1041 (C-O).; HR ESMS *m/z* 461.0838 [M-Na]+. Calc. for $C_{23}H_{23}N_2O_2Br$ 461.0841.

1-(3-Bromophenyl)-3-(3-(3-(4-methoxyphenyl)propyl)phenyl)urea (C.10). Yield 34%; white solid; m.p. 141-144ºC; ^{1}H NMR (400 MHz,DMSO); δ 8.81 (s, 1H), 8.65 (s, 1H), 7.86 (t, J = 1.9 Hz, 1H), 7.32 (t, J = 1.5 Hz, 1H), 7.29 (dd, J = 8.2, 2.0 Hz, 1H), 7.26 – 7.22 (m, 1H), 7.22 – 7.20 (m, 1H), 7.20 – 7.16 (m, 1H), 7.16 – 7.13 (m, 1H), 7.12 (d, J = 8.7 Hz, 2H), 6.84 (d, J = 8.7 Hz, 2H), 6.81 (s, 1H), 3.72 (s, 3H), 2.55 (t, J = 7.6 Hz, 4H), 1.90 – 1.78 (m, 2H).; ^{13}C NMR (100 MHz, DMSO) δ 157.34 (C), 152.31 (C), 142.59 (C), 141.41 (C), 139.31 (C), 133.73 (C), 130.62 (CH), 129.15 (2CH), 128.63 (CH), 124.24 (CH), 122.14 (CH), 121.67 (C), 120.34 (CH), 118.24 (CH), 116.97 (CH), 115.89 (CH), 113.67 (2CH), 54.91 (CH3), 34.72 (CH2), 33.76 (CH2), 32.81 (CH2).; IR $_{vmax}$ (cm-1) 3296 (N-H), 1637 (C=O), 1554 (N-H amide), 1508 (N-C=O), 1244, 1037 (C-O).; HR ESMS *m/z* 461.0839 [M-Na]+. Calc. for $C_{23}H_{23}N_2O_2Br$ 461.0841.

1-(2-Bromophenyl)-3-(3-(3-(4-methoxyphenyl)propyl)phenyl)urea (C.11). Yield 64%; white solid; m.p. 137-140ºC; ^{1}H NMR (400 MHz,DMSO); δ 9.40 (s, 1H), 8.09 (s, 1H), 8.07 (dd, J = 8.3, 1.6 Hz, 1H), 7.61 (dd, J = 8.0, 1.5 Hz, 1H), 7.37 – 7.30 (m, 2H), 7.29 – 7.23 (m, 1H), 7.20 (t, J = 7.7 Hz, 1H), 7.12 (d, J = 8.7 Hz, 2H), 6.97 (ddd, J = 8.0, 7.4, 1.6 Hz, 1H), 6.84 (d, J = 8.7 Hz, 2H), 6.83 – 6.80 (m, 1H), 3.72 (s, 3H), 2.55 (td, J = 7.9, 3.6 Hz, 4H), 1.91 – 1.78 (m, 2H).; ^{13}C NMR (100 MHz, DMSO)) δ 157.34 (C), 152.12 (C), 142.65 (C), 139.41 (C), 137.07 (C), 133.73 (C), 132.41 (CH), 129.14 (2CH),

128.71 (CH), 127.99 (CH), 123.95 (CH), 122.17 (2CH), 118.05 (CH), 115.70 (CH), 113.67 (2CH), 112.95 (C), 54.91 (CH3), 34.72 (CH2), 33.76 (CH2), 32.80 (CH2).; IR $_{vmax}$ (cm-1) 3270 (N-H), 1643 (C=O), 1558 (N-H amide), 1508 (N-C=O), 1249, 1041 (C-O).; HR ESMS *m/z* 461.0842 [M-Na]+. Calc. for $C_{23}H_{23}N_2O_2Br$ 461.0841.

1-(4-Methoxyphenyl)-3-(3-(3-(4-methoxyphenyl)propyl)phenyl)urea (C.12). Yield 34%; white solid; m.p. 181-183ºC; ^{1}H NMR (400 MHz,DMSO); δ 8.48 (s, 1H), 8.40 (s, 1H), 7.34 (d, J = 9.1 Hz, 2H), 7.30 (t, J = 1.7 Hz, 1H), 7.25 – 7.20 (m, 1H), 7.19 – 7.14 (m, 1H), 7.12 (d, J = 8.7 Hz, 2H), 6.86 (d, J = 9.0 Hz, 3H), 6.84 (d, J = 8.6 Hz, 3H), 6.78 (d, J = 7.4 Hz, 1H), 3.72 (s, 3H), 3.71 (s, 3H), 2.59 – 2.52 (m, 4H), 1.88 – 1.77 (m, 2H).; ^{13}C NMR (100 MHz, DMSO)) δ 154.42 (C), 152.68 (C), 142.50 (C), 139.81 (C), 133.75 (C), 132.70 (C), 129.14 (2CH), 128.57 (CH), 121.65 (CH), 119.97 (2CH), 117.92 (CH), 115.58 (CH), 113.94 (2CH), 113.67 (2CH), 55.14 (CH3), 54.91 (CH3), 34.74 (CH2), 33.77 (CH2), 32.82 (CH2).; IR $_{vmax}$ (cm-1) 3289 (N-H), 1633 (C=O), 1554 (N-H amide), 1508 (N-C=O), 1238,1179,1021 (C-O).; HR ESMS *m/z* 391.2024 [M-H]+. Calc. for $C_{24}H_{26}N_2O_3$ 391.2022.

1-(3-Methoxyphenyl)-3-(3-(3-(4-methoxyphenyl)propyl)phenyl)urea (C.13). Yield 90%; white solid; m.p. 107-110ºC; ^{1}H NMR (400 MHz,DMSO) δ 8.62 (s, 1H), 8.56 (s, 1H), 7.31 (t, *J* = 1.6 Hz, 1H), 7.26 – 7.20 (m, 1H), 7.20 – 7.14 (m, 3H), 7.12 (d, *J* = 8.7 Hz, 2H), 6.92 (dd, *J* = 8.1, 1.9 Hz, 1H), 6.84 (d, *J* = 8.7 Hz, 2H), 6.81 (d, *J* = 7.4 Hz, 1H), 6.54 (ddd, *J* = 8.2, 2.5, 0.8 Hz, 1H), 3.73 (s, 3H), 3.72 (s, 3H), 2.58 – 2.52 (m, 4H), 1.84 (dq, *J* = 15.3, 7.7 Hz, 2H).; ^{13}C NMR (100 MHz, DMSO)) δ 159.67 (C), 157.34 (C), 152.41 (C), 142.57 (C), 140.92 (C), 139.55 (C), 133.74 (C), 129.46 (CH), 129.14 (2CH), 128.61 (CH), 121.90 (CH), 118.05 (CH), 115.72 (CH), 113.67 (2CH), 110.45 (CH), 107.20 (CH), 103.88 (CH), 54.91 (CH3), 54.89 (CH3), 34.74 (CH2), 33.77 (CH2), 32.83 (CH2).; IR $_{vmax}$ (cm-1) 3282 (N-H), 1640 (C=O), 1551 (N-H amide), 1508 (N-C=O), 1241,1153,1038 (C-O).; HR ESMS *m/z* 391.2024 [M-H]+. Calc. for $C_{24}H_{26}N_2O_3$ 391.2022.

1-(2-Methoxyphenyl)-3-(3-(3-(4-methoxyphenyl)propyl)phenyl)urea (C.14). Yield 78%; white solid; m.p. 82-85ºC; ^{1}H NMR (400 MHz,DMSO) δ 9.24 (s, 1H), 8.19 (s, 1H), 8.14 (dd, J = 7.8, 1.8 Hz, 1H), 7.33 (t, J = 1.6 Hz, 1H), 7.27 – 7.22 (m, 1H), 7.18 (t, J =

7.7 Hz, 1H), 7.12 (d, J = 8.6 Hz, 2H), 7.01 (dd, J = 8.0, 1.6 Hz, 1H), 6.94 (td, J = 7.7, 1.8 Hz, 1H), 6.88 (td, J = 8.1, 2.1 Hz, 1H), 6.84 (d, J = 8.7 Hz, 2H), 6.80 (d, J = 7.5 Hz, 1H), 3.87 (s, 3H), 3.72 (s, 3H), 2.55 (t, J = 7.6 Hz, 4H), 1.90 – 1.76 (m, 2H).; ^{13}C NMR (100 MHz, DMSO)) δ 157.34 (C), 152.35 (C), 147.59 (C), 142.58 (C), 139.79 (C), 133.75 (C), 129.14 (2CH), 128.70 (C), 128.65 (CH), 121.77 (CH), 121.67 (CH), 120.50 (CH), 118.25 (CH), 117.76 (CH), 115.42 (CH), 113.67 (2CH), 110.68 (CH), 55.73 (CH3), 54.90 (CH3), 34.76 (CH2), 33.78 (CH2), 32.82 (CH2).; IR $_{vmax}$ (cm-1) 3302 (N-H), 1647 (C=O), 1541 (N-H amide), 1515 (N-C=O), 1244, 1218, 1172,1021 (C-O).; HR ESMS *m/z* 391.2015 [M-H]+. Calc. for $C_{24}H_{26}N_2O_3$ 391.2022.

Graphical NMR spectra

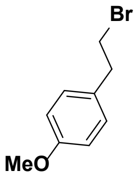

C.16

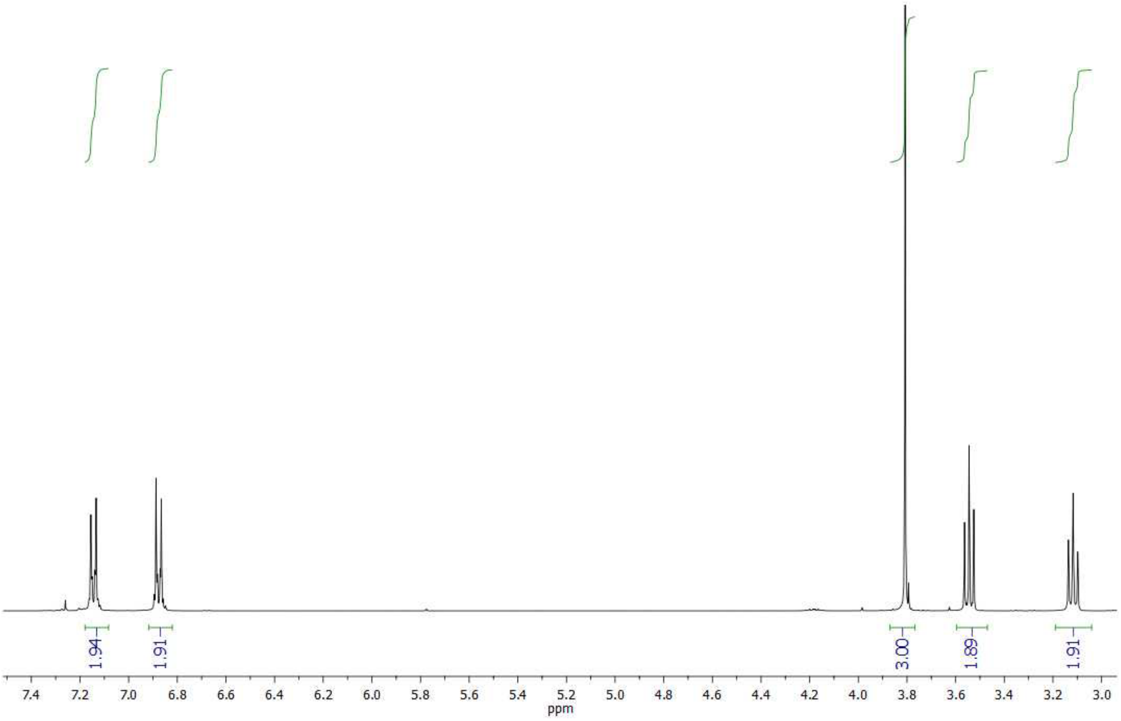

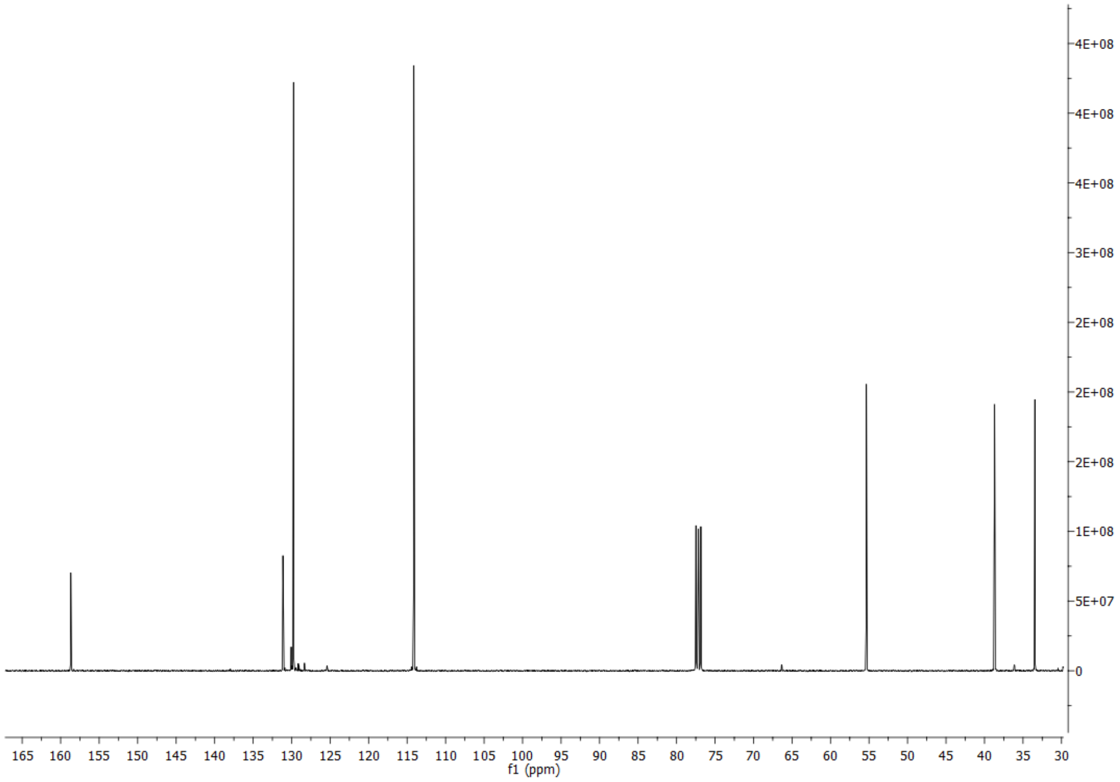

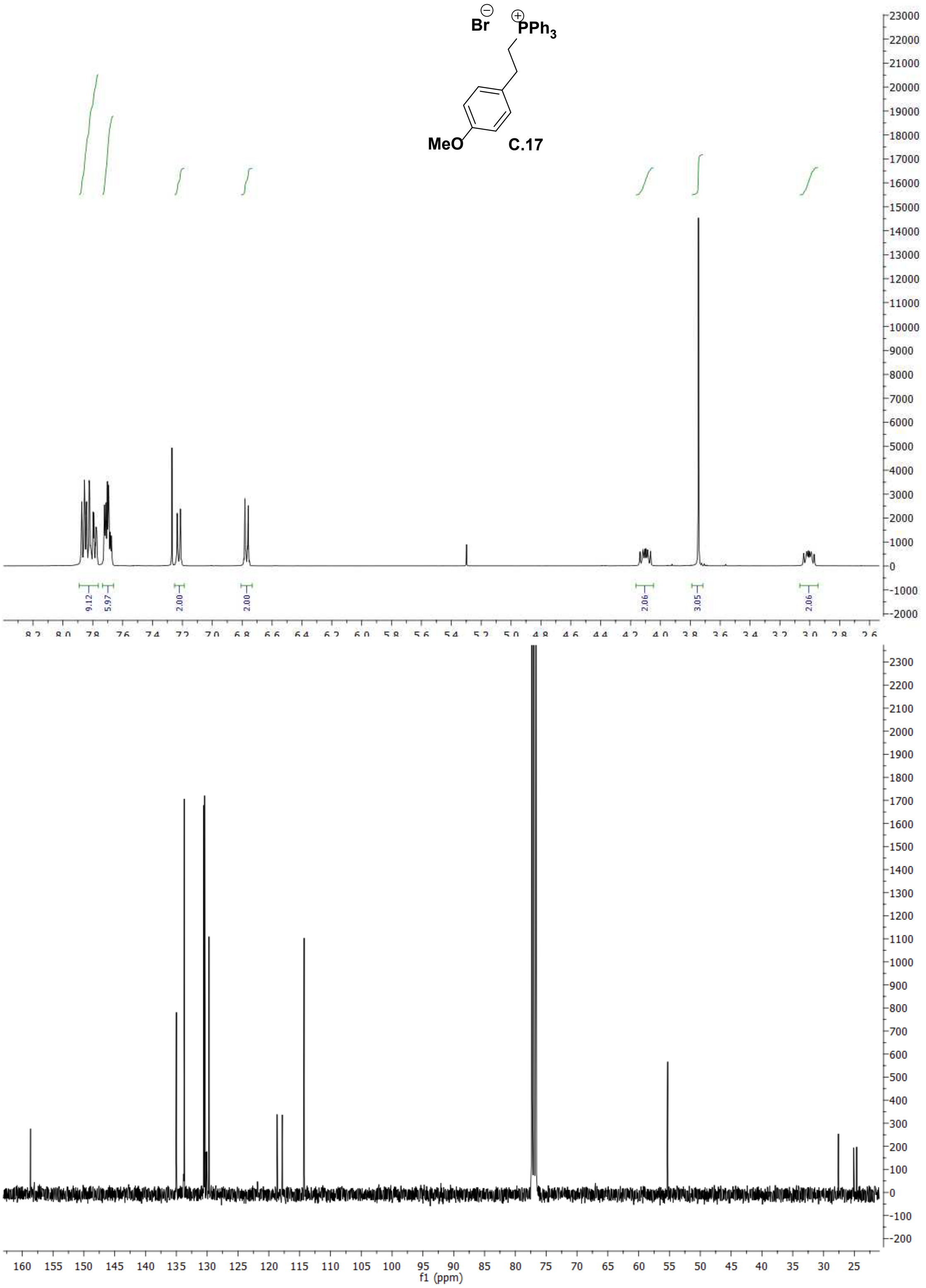
Br
PPh3
MeO
C.17
9.12
5.97
2.00
2.00
2.06
3.05
2.06
f1 (ppm)

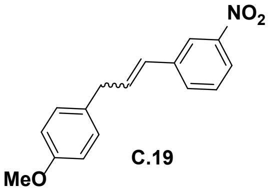

C.19

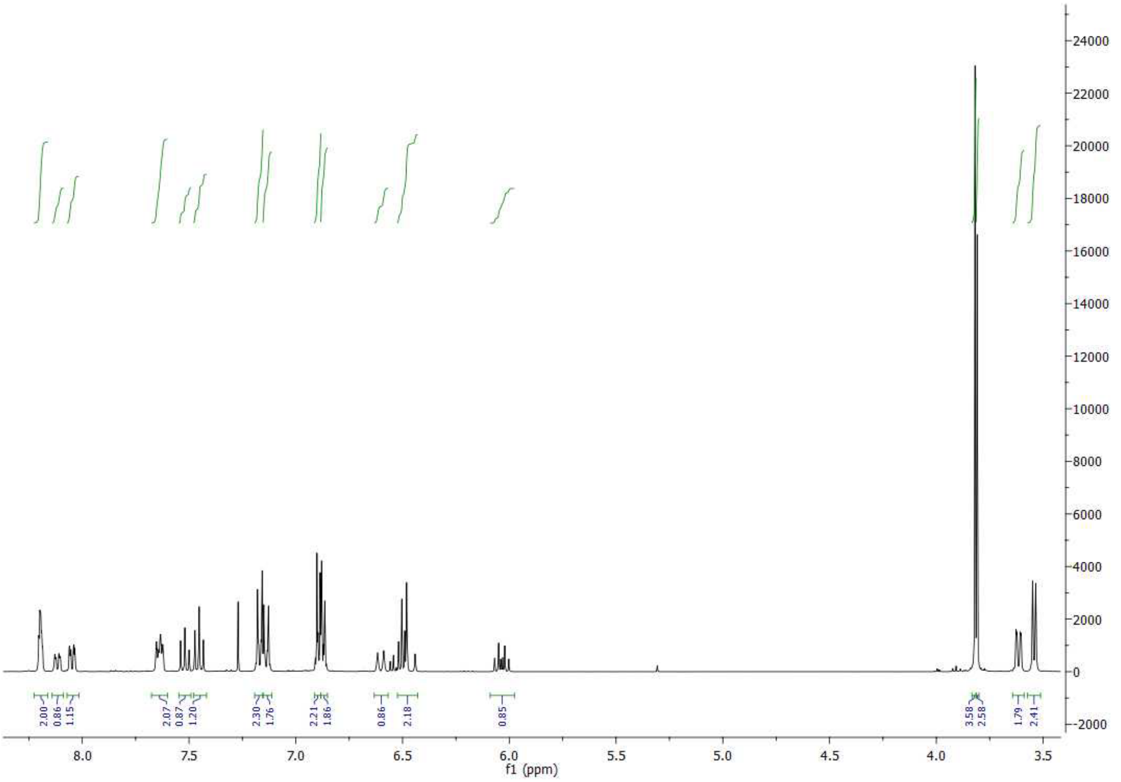

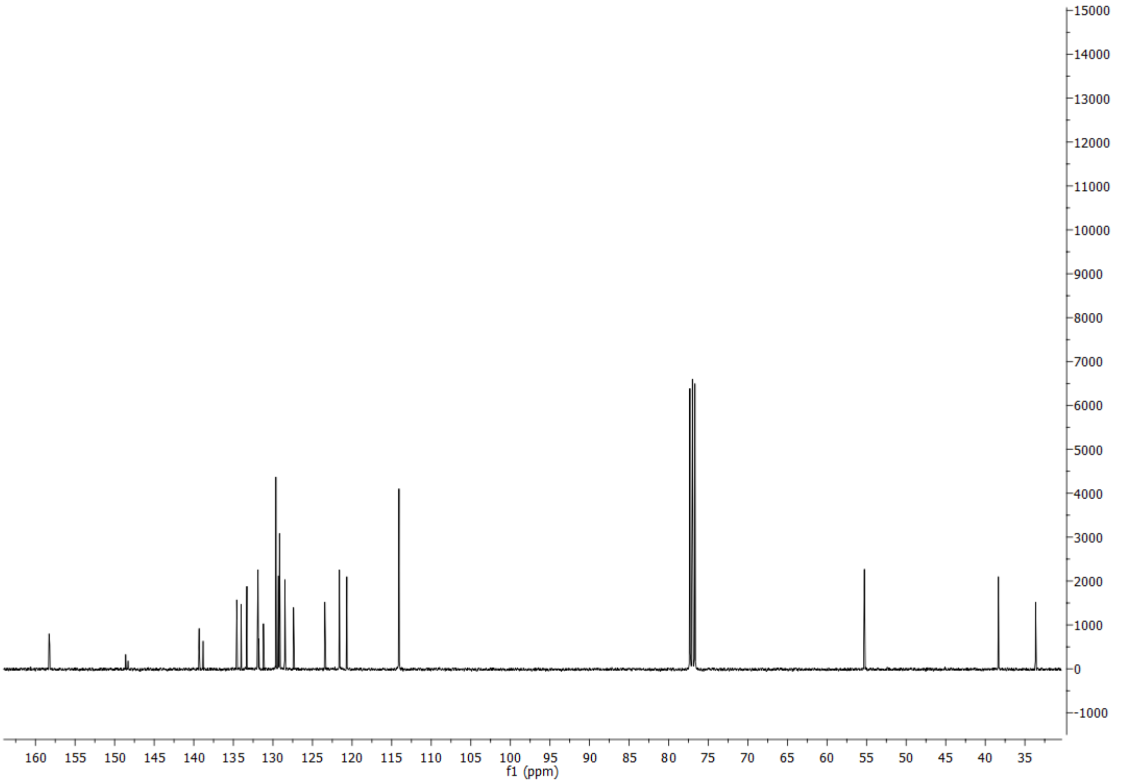

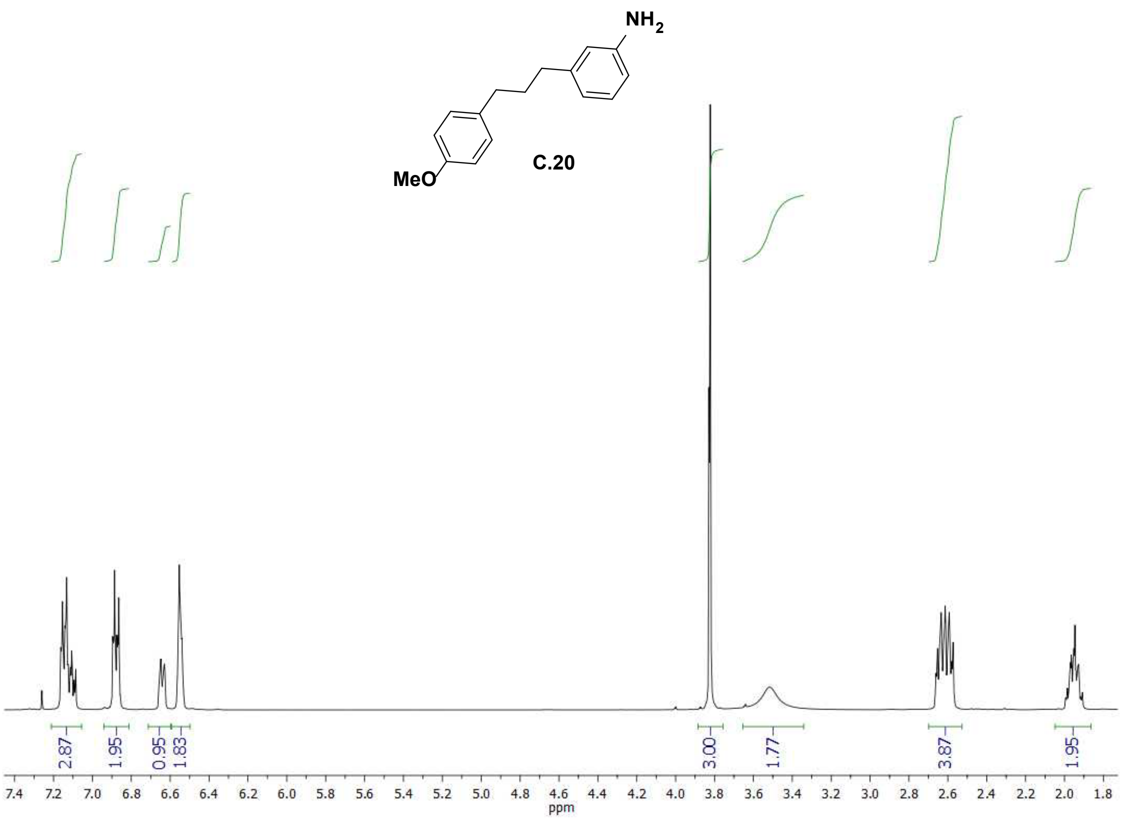
NH2
MeO
C.20
2.87
1.95
0.95
1.83
3.00
1.77
3.87
1.95
7.4 7.2 7.0 6.8 6.6 6.4 6.2 6.0 5.8 5.6 5.4 5.2 5.0 4.8 4.6 4.4 4.2 4.0 3.8 3.6 3.4 3.2 3.0 2.8 2.6 2.4 2.2 2.0 1.8
ppm

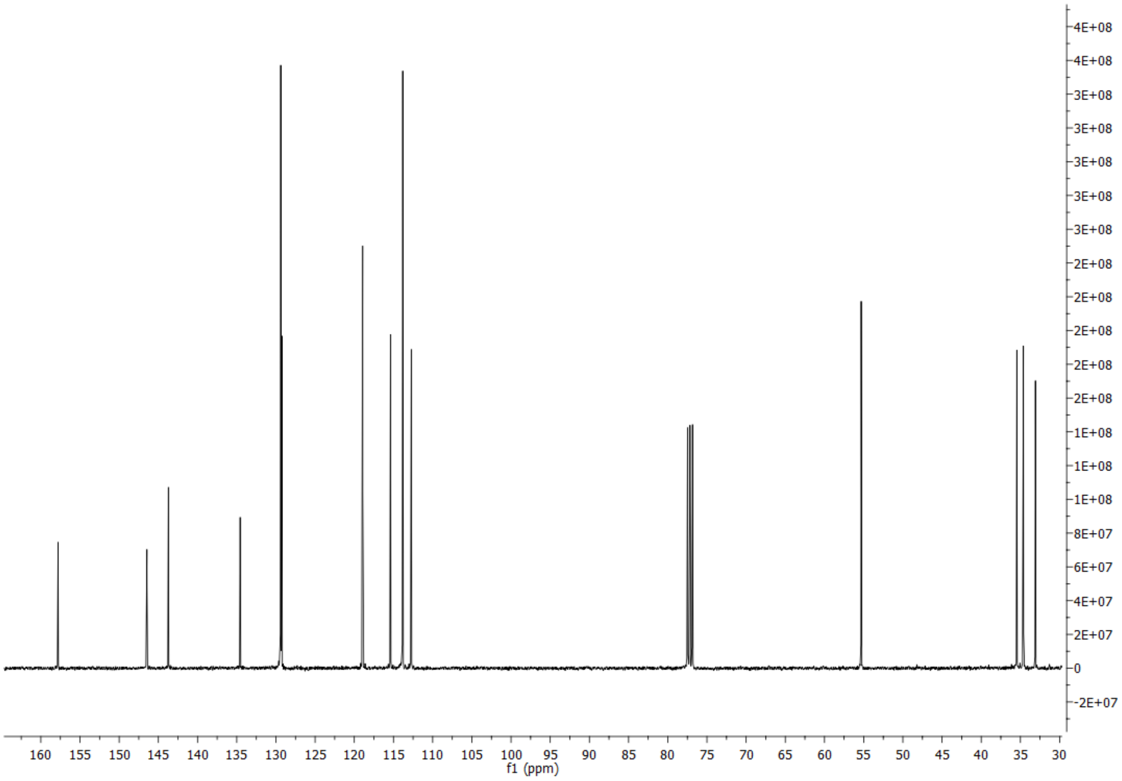
4E+08
4E+08
3E+08
3E+08
3E+08
3E+08
3E+08
2E+08
2E+08
2E+08
2E+08
2E+08
1E+08
1E+08
1E+08
8E+07
6E+07
4E+07
2E+07
0
-2E+07
160 155 150 145 140 135 130 125 120 115 110 105 100 95 90 85 80 75 70 65 60 55 50 45 40 35 30
f1 (ppm)

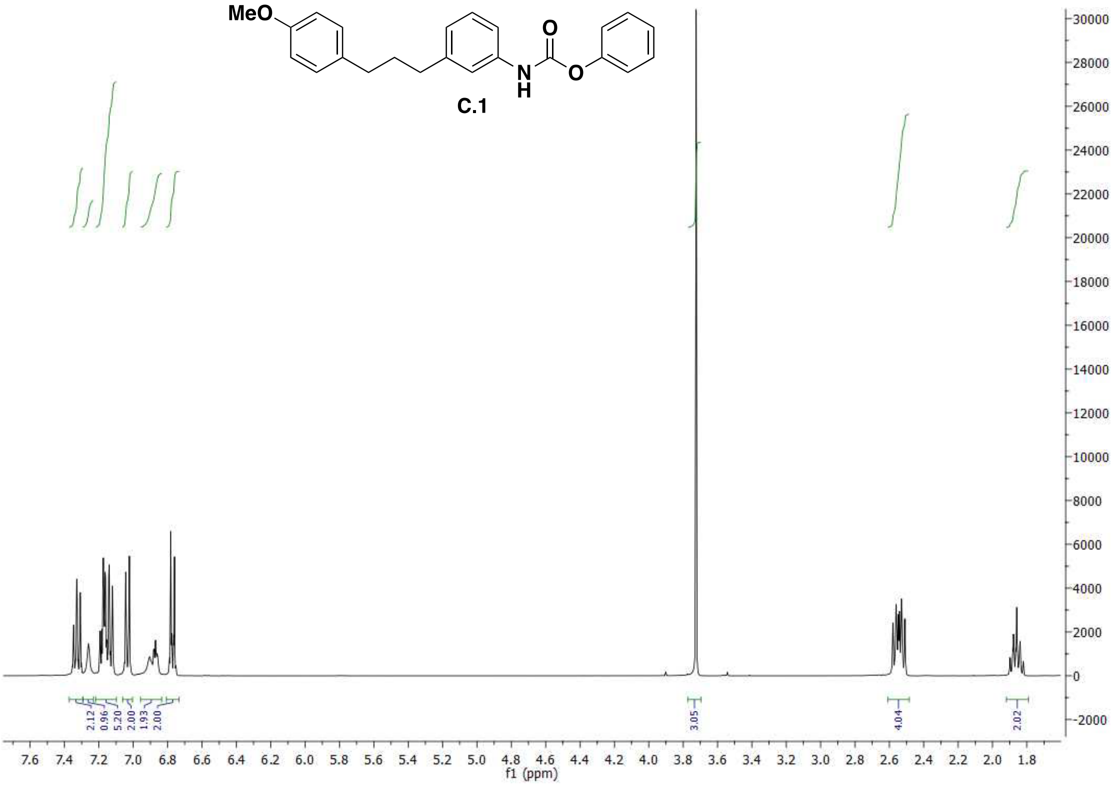
MeO
O
N
H
O

C.1

2.12
0.96
5.20
2.00
1.93
2.00
3.05
4.04
2.02
7.6 7.4 7.2 7.0 6.8 6.6 6.4 6.2 6.0 5.8 5.6 5.4 5.2 5.0 4.8 4.6 4.4 4.2 4.0 3.8 3.6 3.4 3.2 3.0 2.8 2.6 2.4 2.2 2.0 1.8
f1 (ppm)
30000
28000
26000
24000
22000
20000
18000
16000
14000
12000
10000
8000
6000
4000
2000
0
-2000

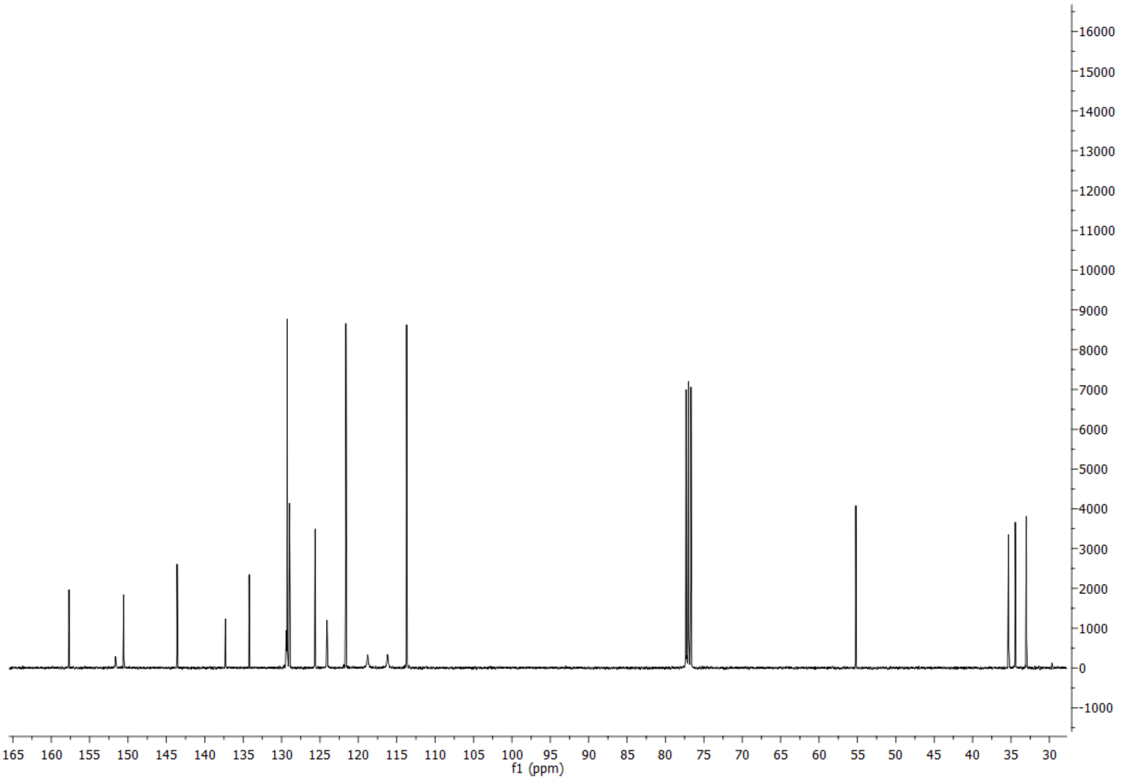
165 160 155 150 145 140 135 130 125 120 115 110 105 100 95 90 85 80 75 70 65 60 55 50 45 40 35 30
f1 (ppm)
16000
15000
14000
13000
12000
11000
10000
9000
8000
7000
6000
5000
4000
3000
2000
1000
0
-1000

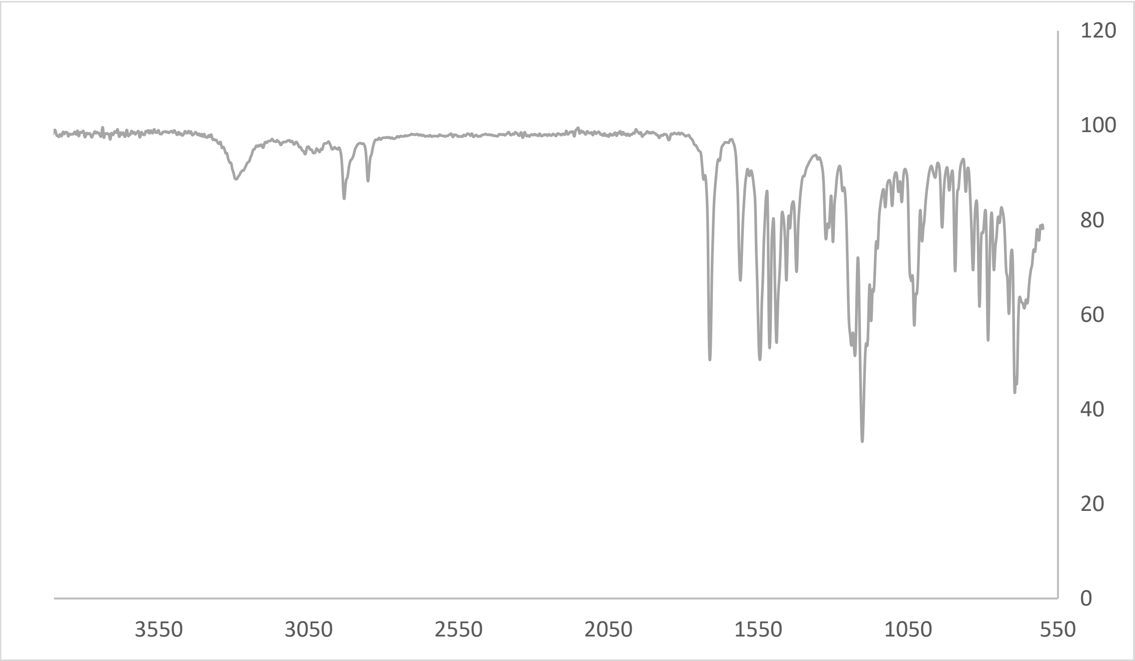
120
100
80
60
40
20
0
3550
3050
2550
2050
1550
1050
550

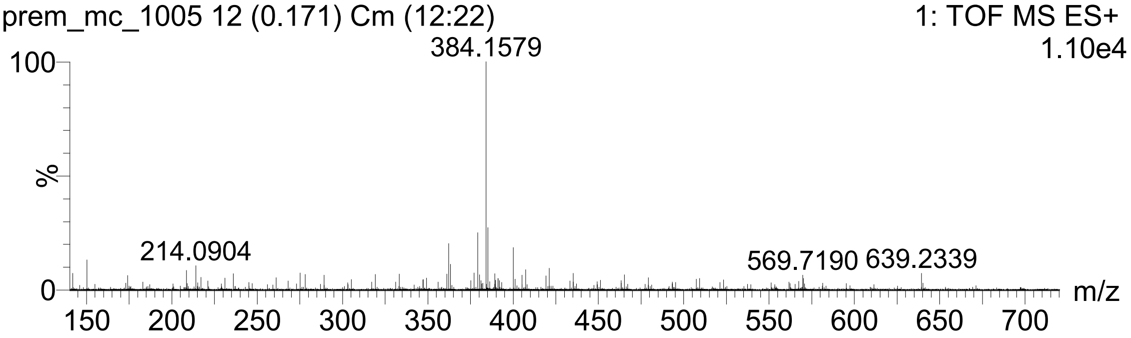
prem_mc_1005 12 (0.171) Cm (12:22)
1: TOF MS ES+
1.10e4
384.1579
100
%
214.0904
569.7190
639.2339
0
m/z
150
200
250
300
350
400
450
500
550
600
650
700

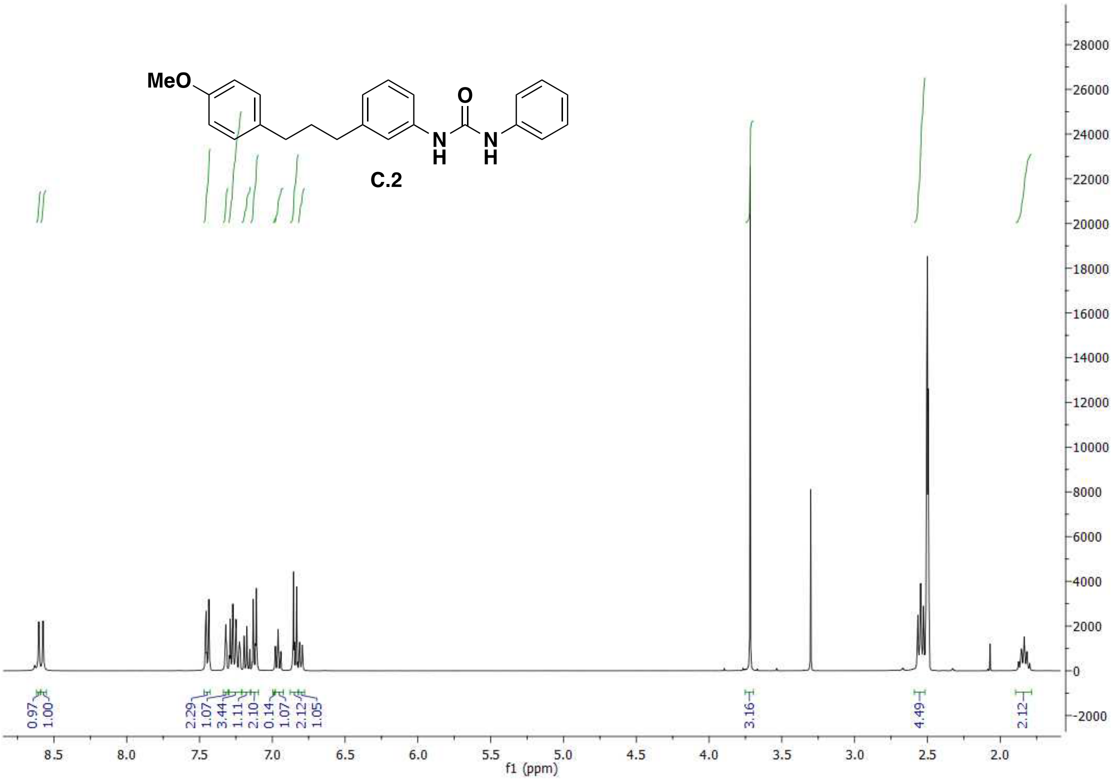
MeO
O
N
H
N
H
C.2
0.97
1.00
2.29
1.07
3.44
1.11
2.10
0.14
1.07
2.12
1.05
3.16
4.49
2.12
f1 (ppm)

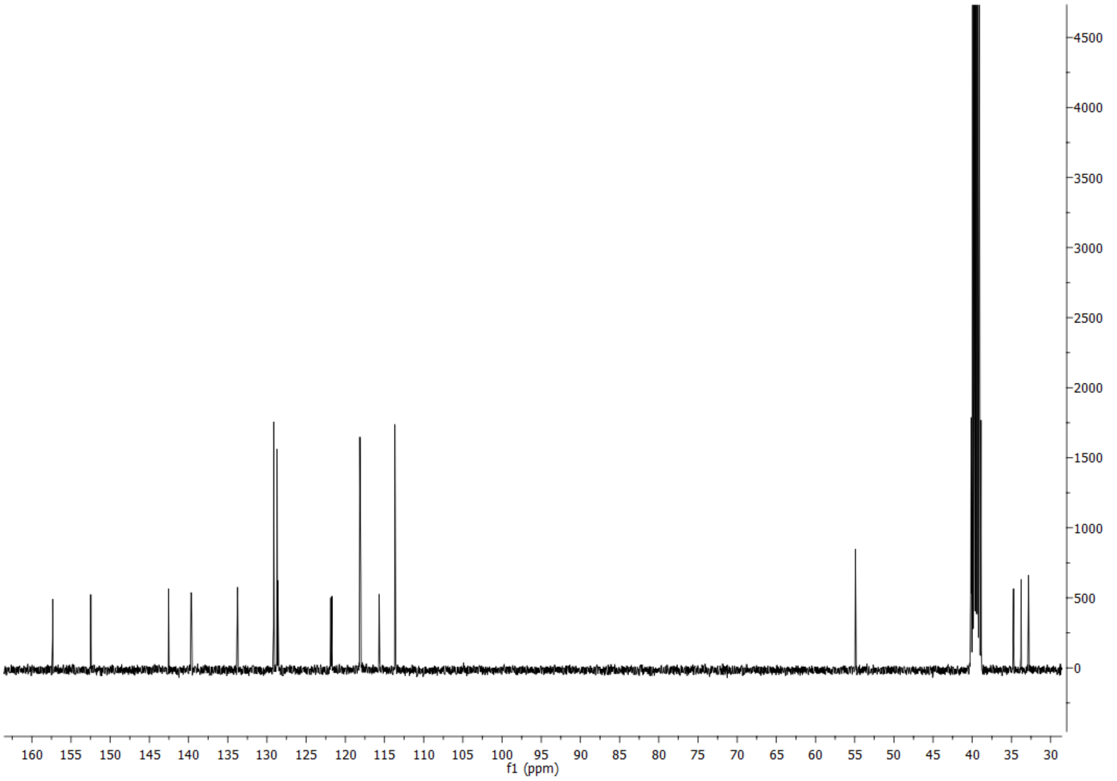
f1 (ppm)

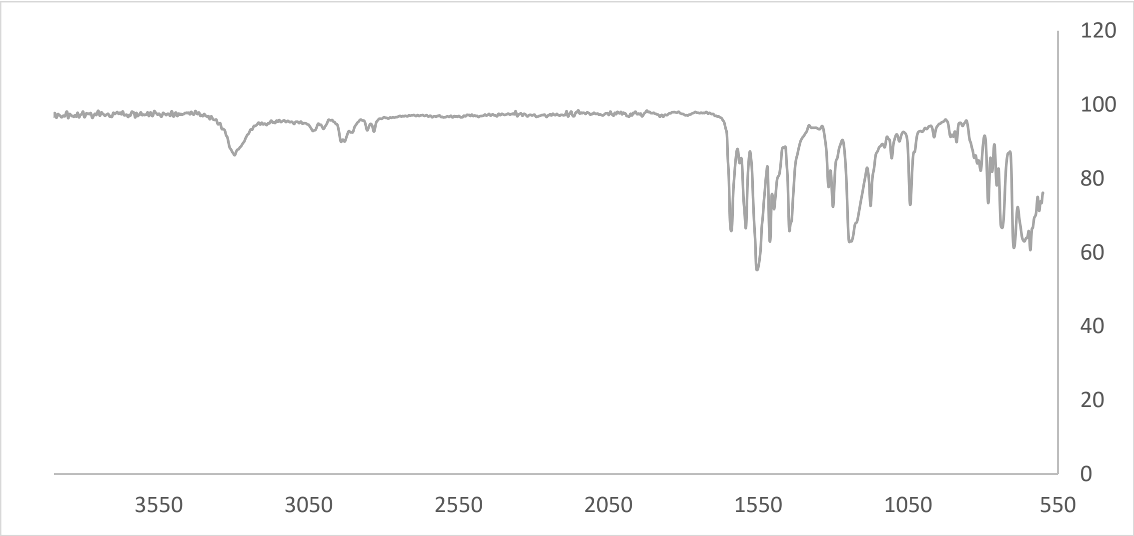
120
100
80
60
40
20
0
3550
3050
2550
2050
1550
1050
550

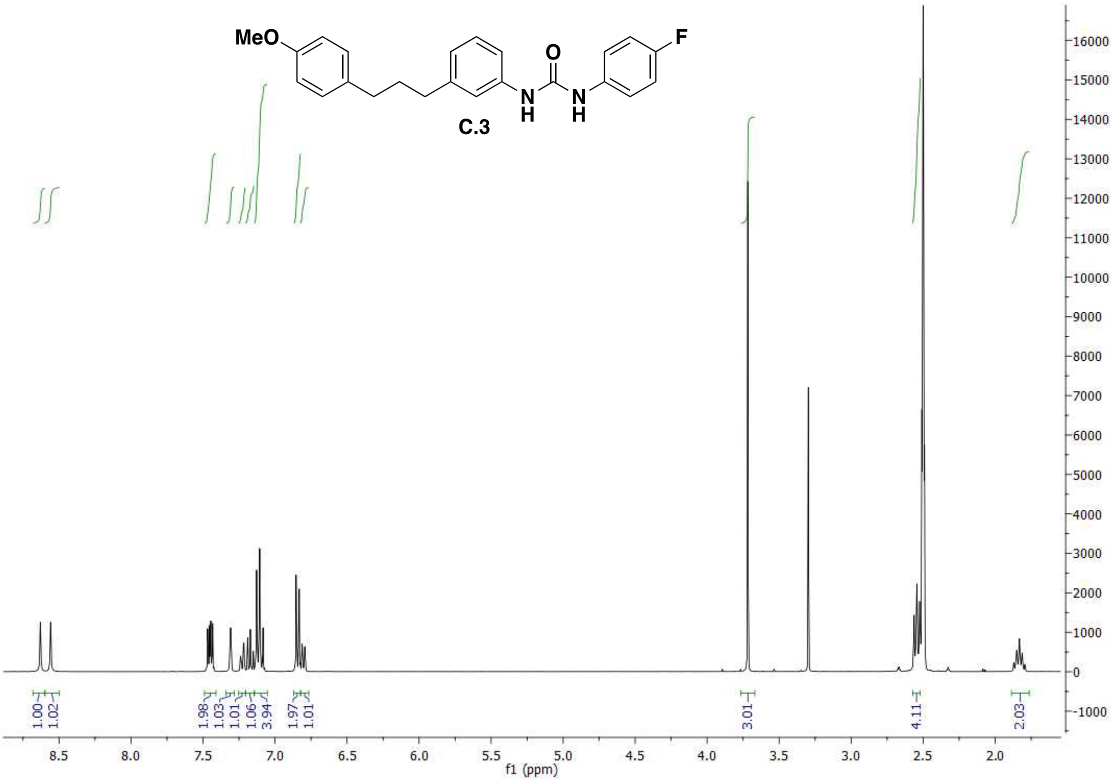
MeO
F
O
N
H
N
H
C.3
1.00
1.02
1.98
1.03
1.01
1.06
3.94
1.97
1.01
3.01
4.11
2.03
f1 (ppm)

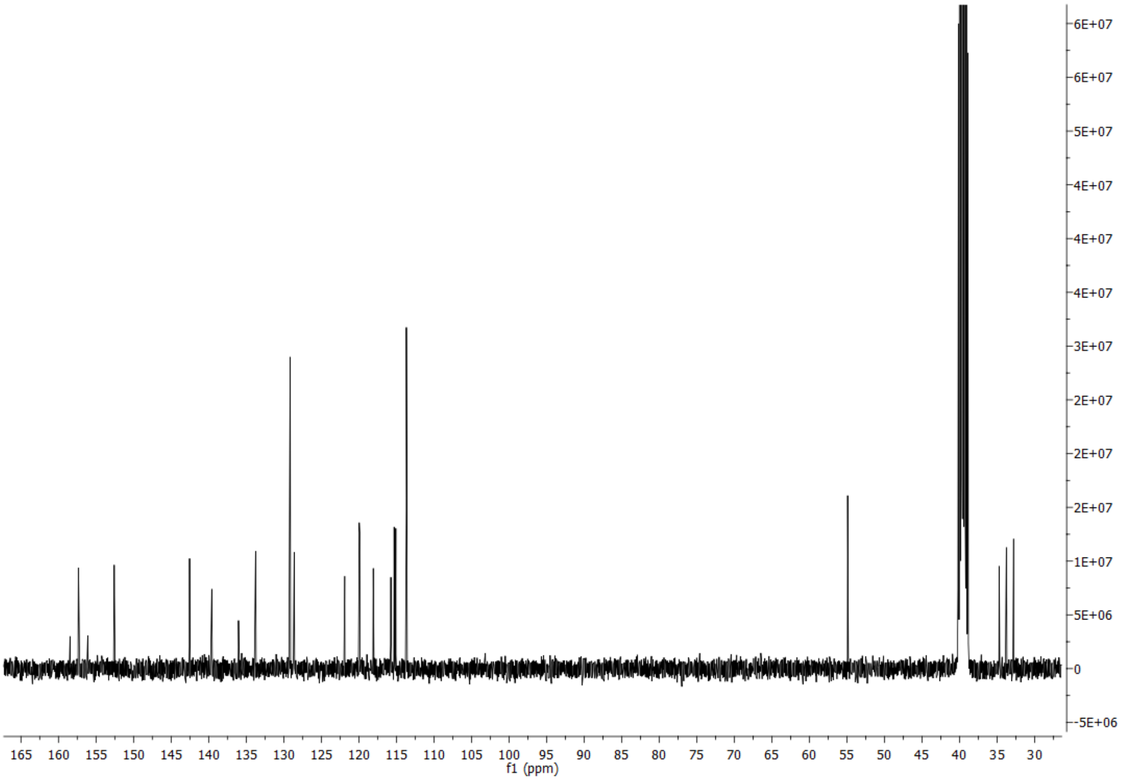
f1 (ppm)

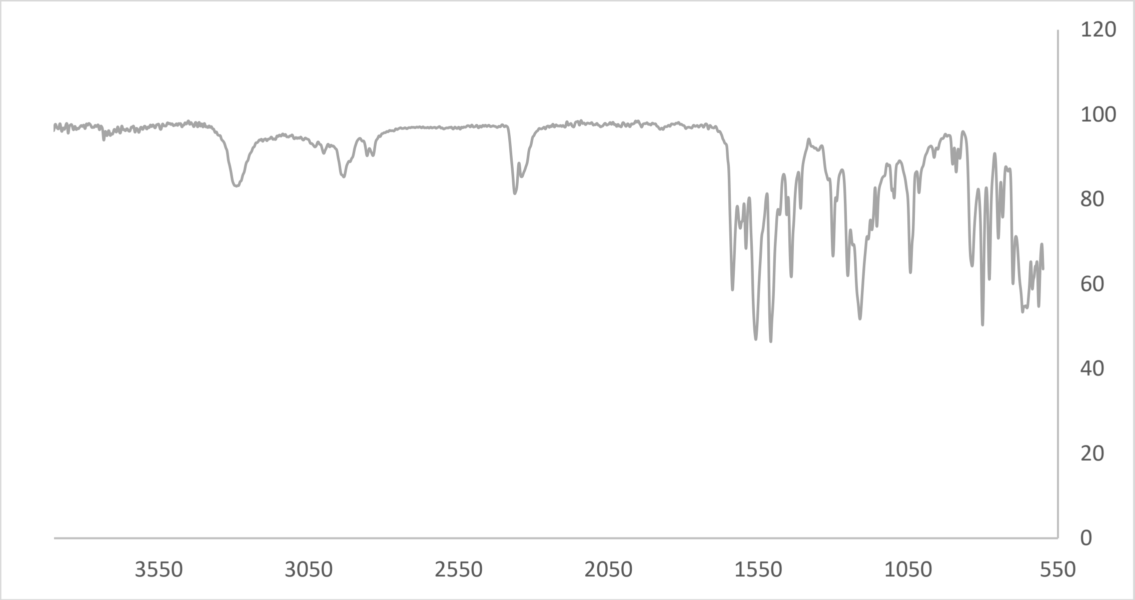
120
100
80
60
40
20
0
3550
3050
2550
2050
1550
1050
550

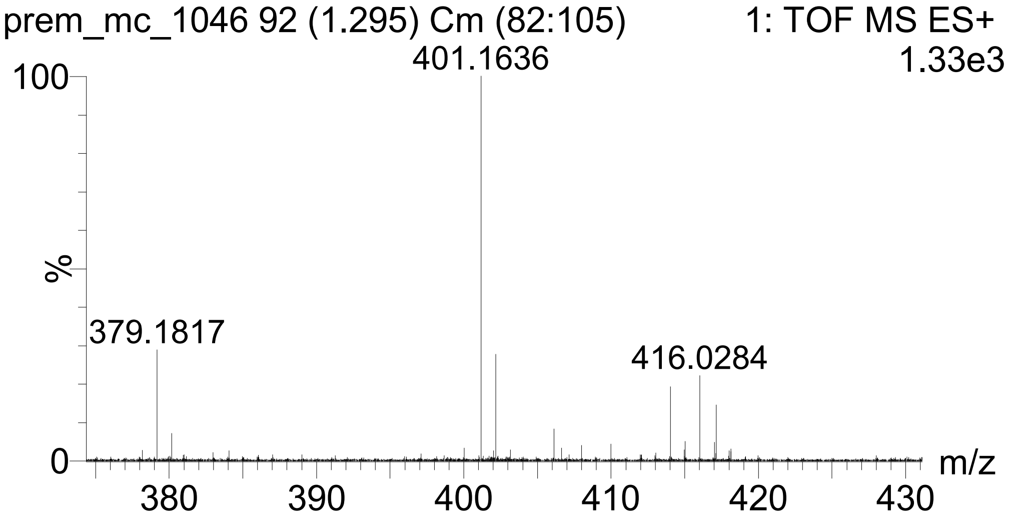
prem_mc_1046 92 (1.295) Cm (82:105)
1: TOF MS ES+
1.33e3
401.1636
100
%
379.1817
416.0284
0
m/z
380
390
400
410
420
430

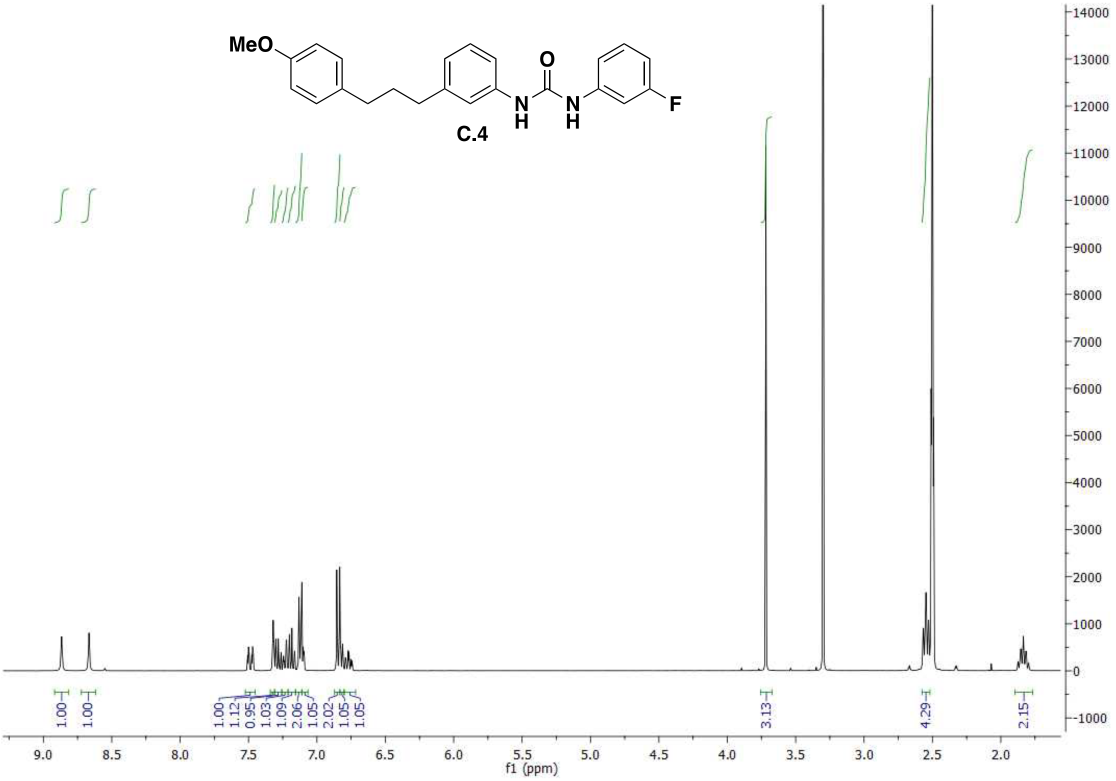
MeO
O
N
H
N
H
F
C.4
f1 (ppm)

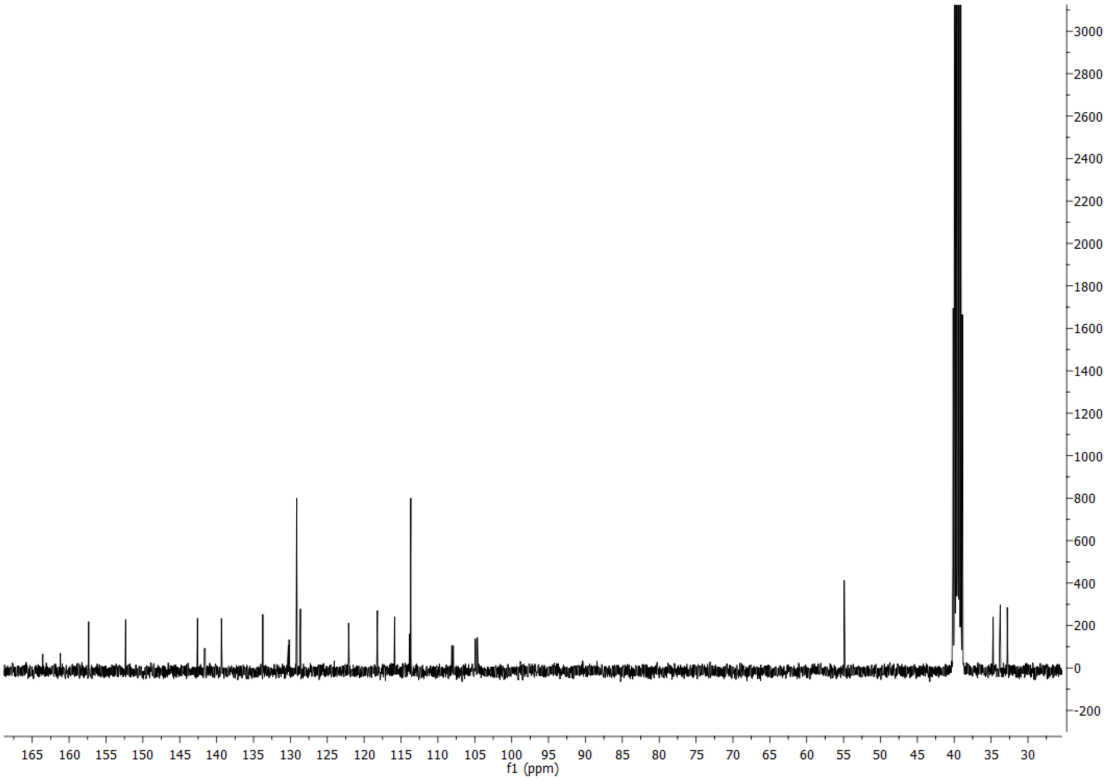
f1 (ppm)

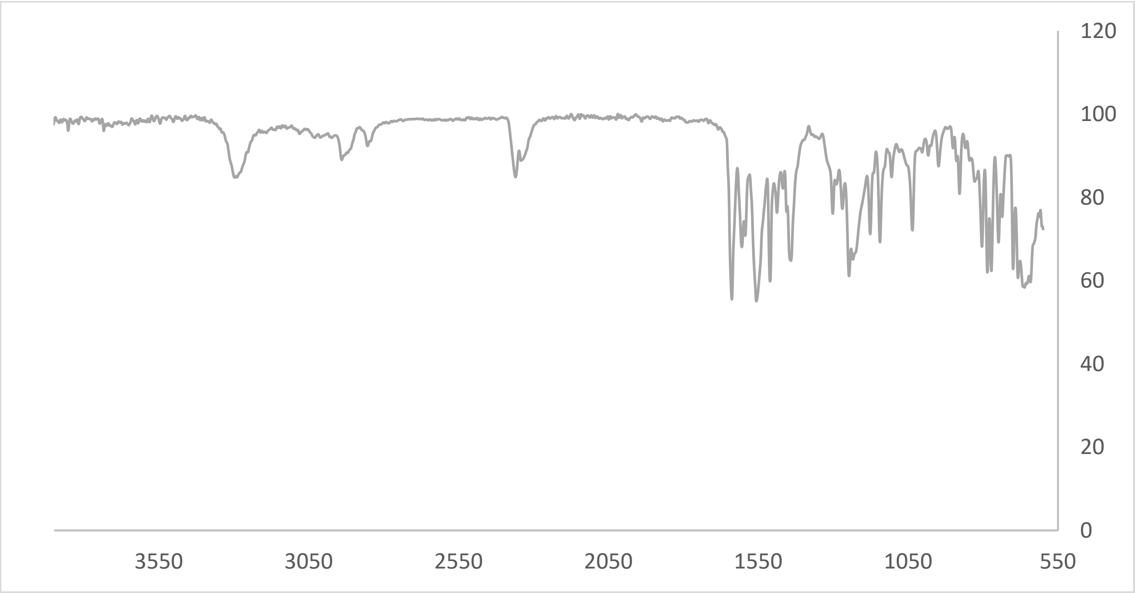
120
100
80
60
40
20
0
3550
3050
2550
2050
1550
1050
550

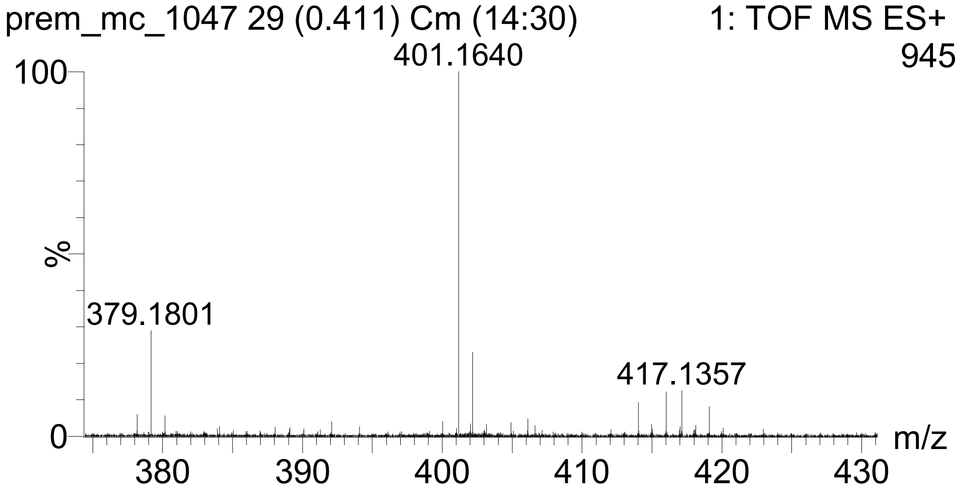
prem_mc_1047 29 (0.411) Cm (14:30)
1: TOF MS ES+
945
401.1640
100
%
379.1801
417.1357
0
m/z
380
390
400
410
420
430

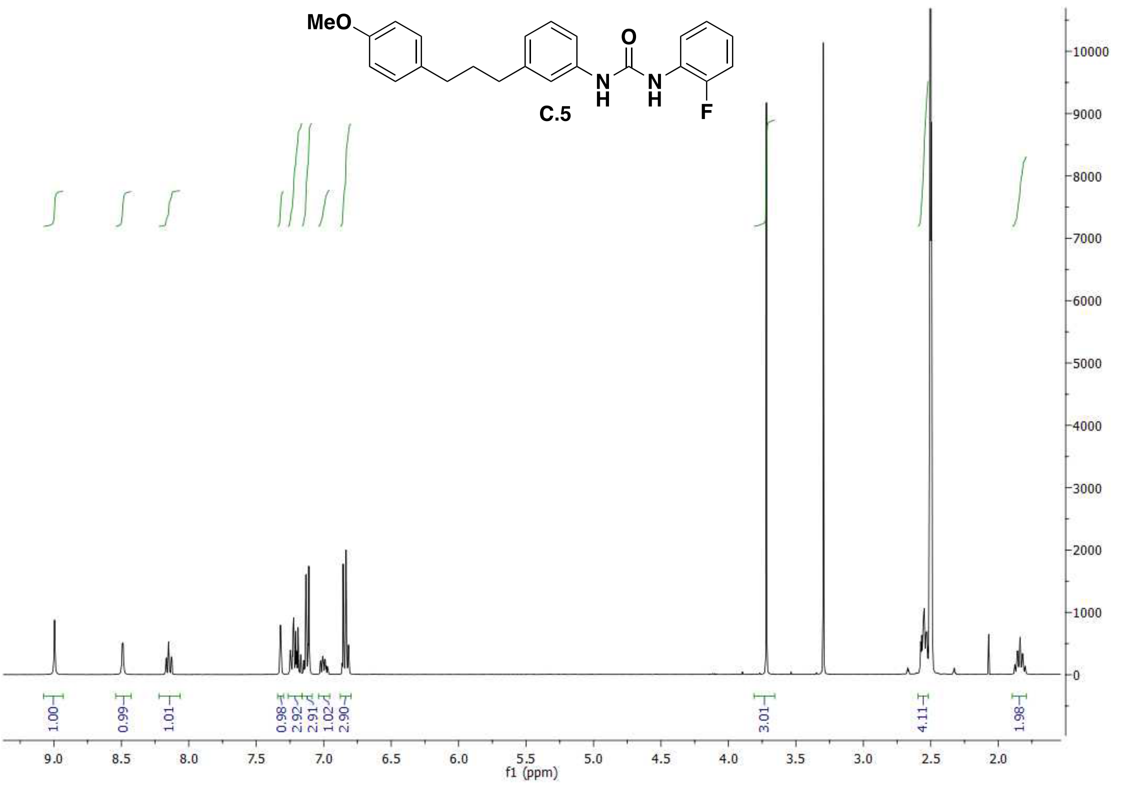
MeO
C.5
f1 (ppm)
1.00
0.99
1.01
0.98
2.92
2.91
1.02
2.90
3.01
4.11
1.98

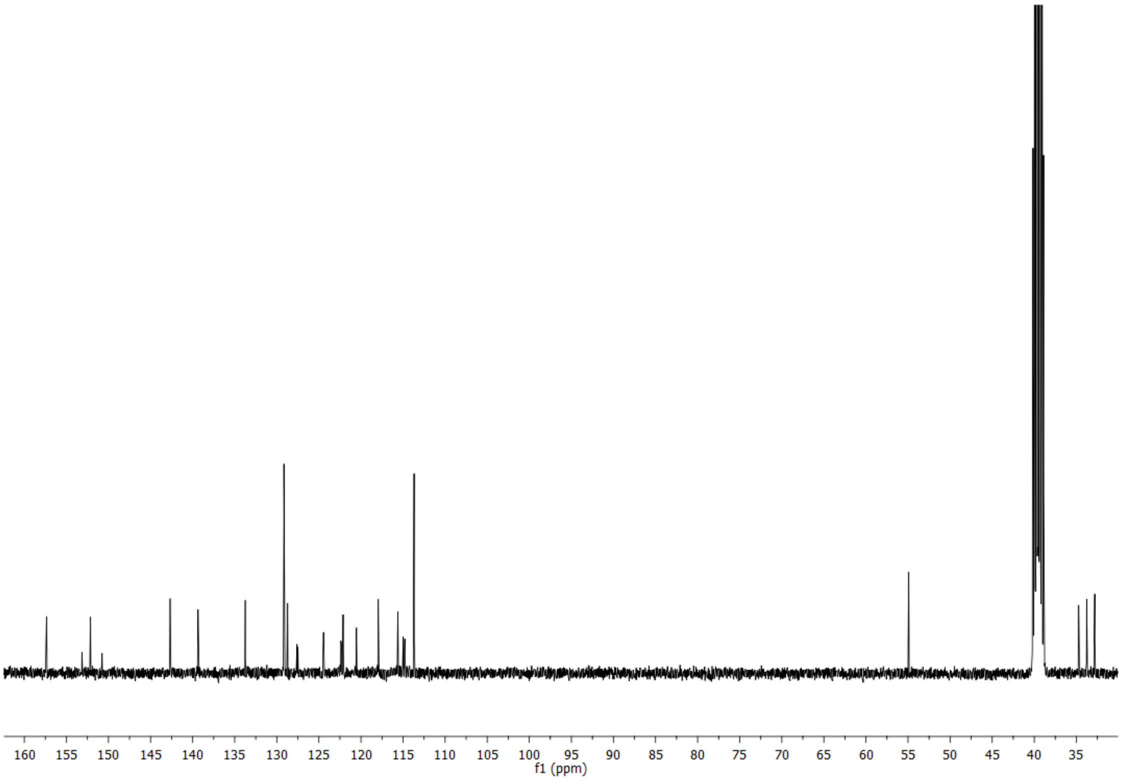
f1 (ppm)

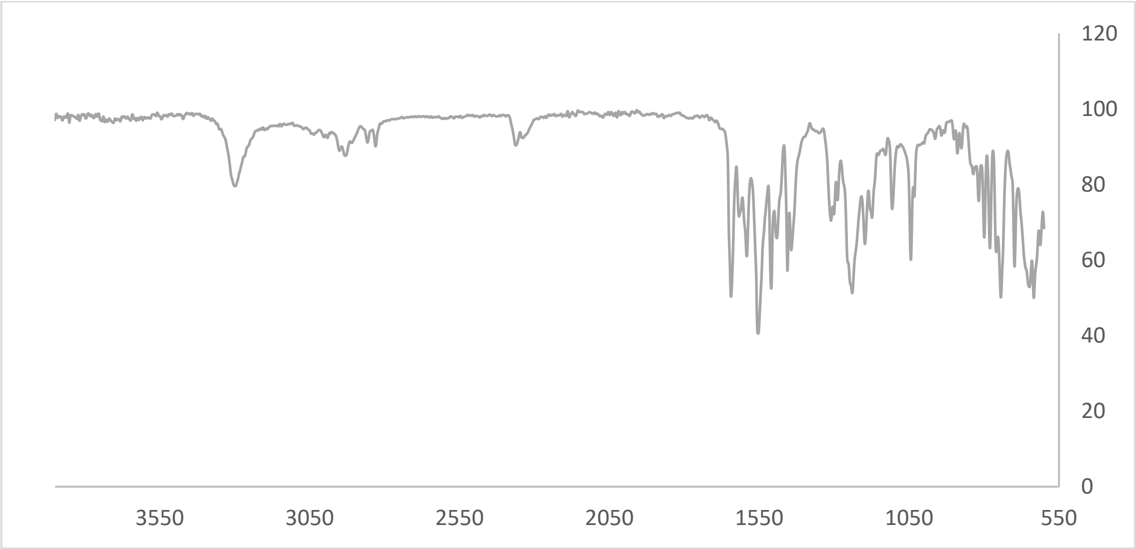
120
100
80
60
40
20
0
3550
3050
2550
2050
1550
1050
550

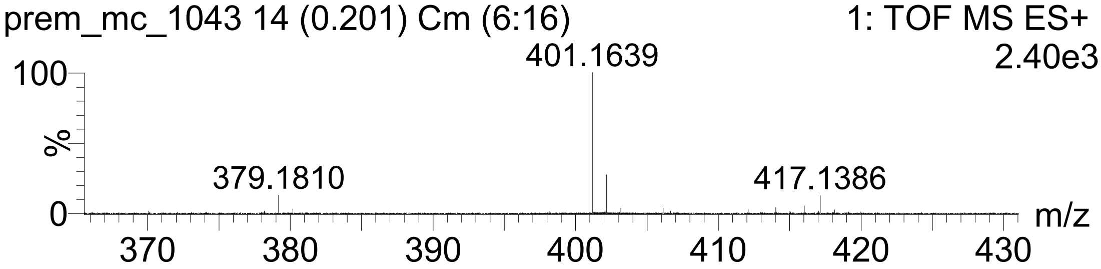
prem_mc_1043 14 (0.201) Cm (6:16)
1: TOF MS ES+
2.40e3
401.1639
100
%
379.1810
417.1386
0
m/z
370
380
390
400
410
420
430

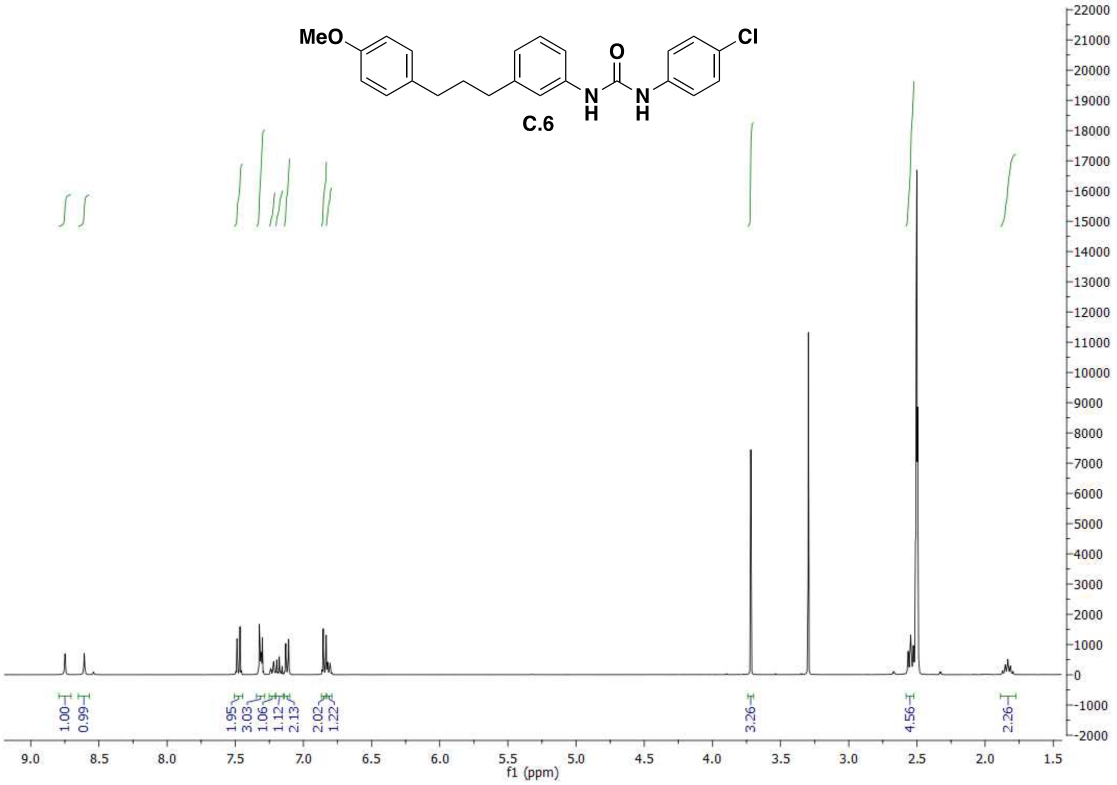
MeO
Cl
O
N
H
N
H
C.6
1.00
0.99
1.95
3.03
1.06
1.12
2.13
2.02
1.22
3.26
4.56
2.26
f1 (ppm)

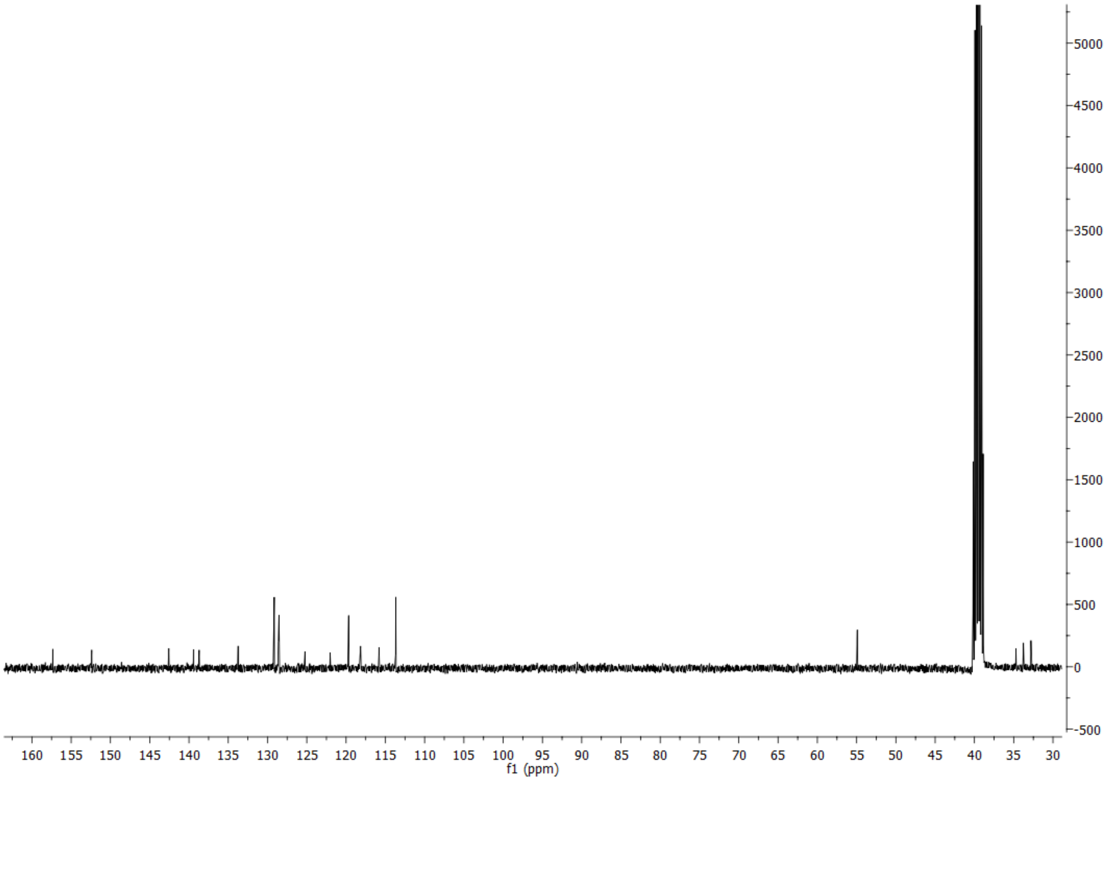
f1 (ppm)

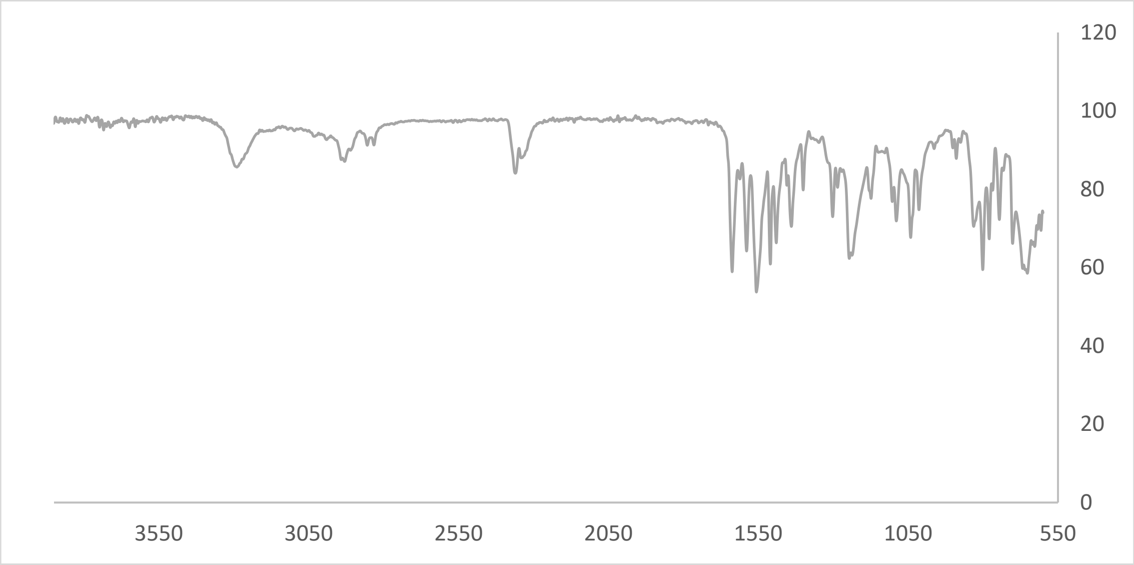
120
100
80
60
40
20
0
3550
3050
2550
2050
1550
1050
550

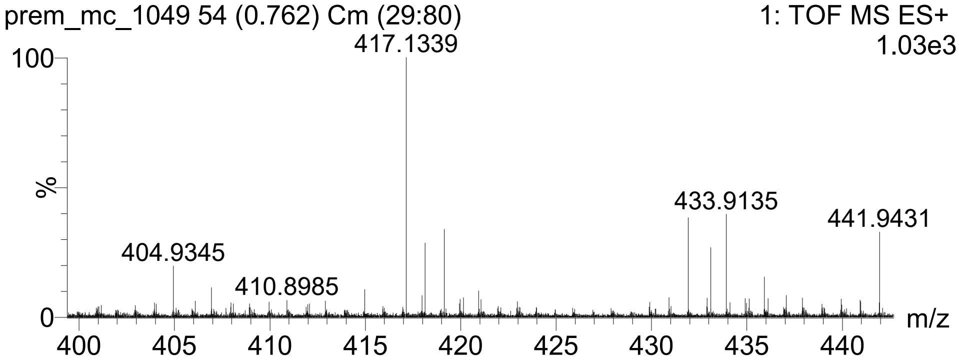
prem_mc_1049 54 (0.762) Cm (29:80)
1: TOF MS ES+
1.03e3
417.1339
100
%
433.9135
441.9431
404.9345
410.8985
0
400
405
410
415
420
425
430
435
440
m/z

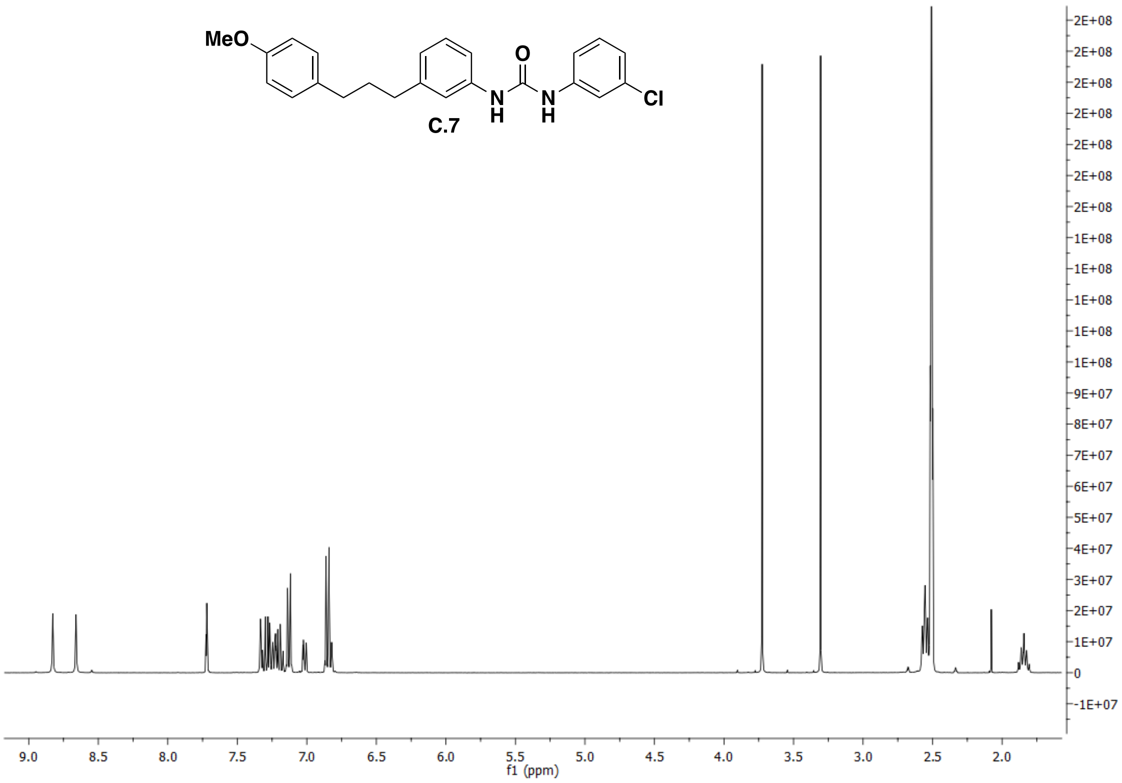

MeO
O
N
H
N
H
Cl
C.7
f1 (ppm)

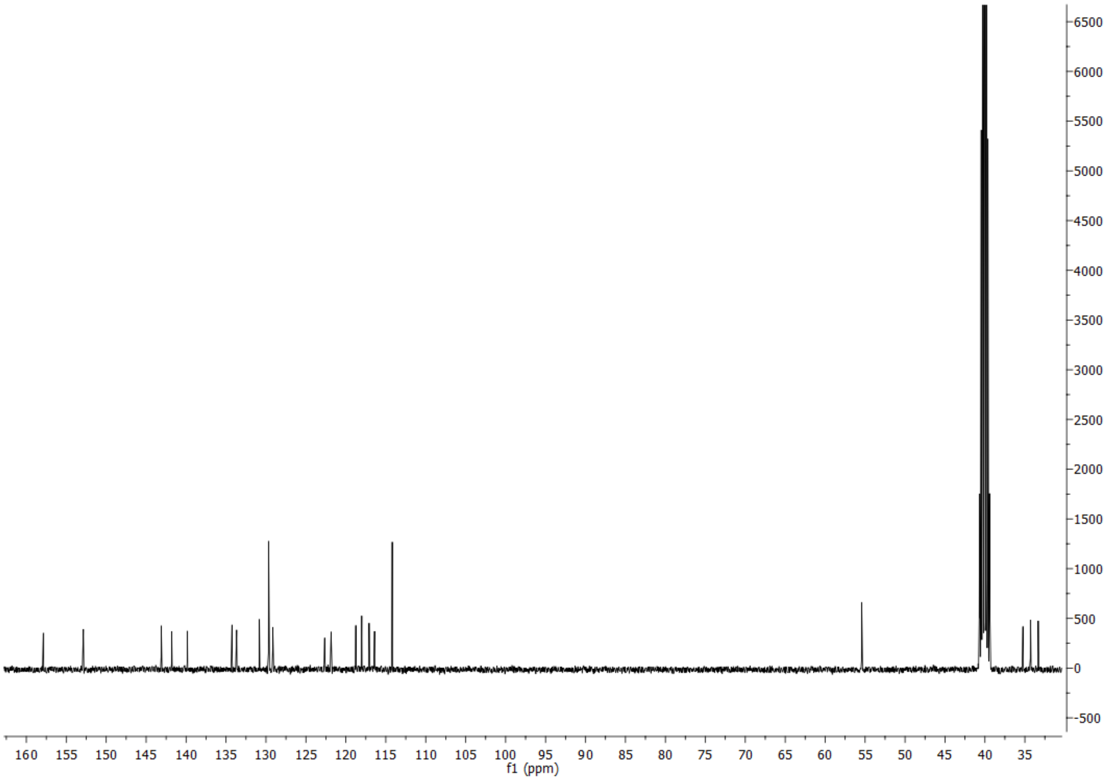

f1 (ppm)

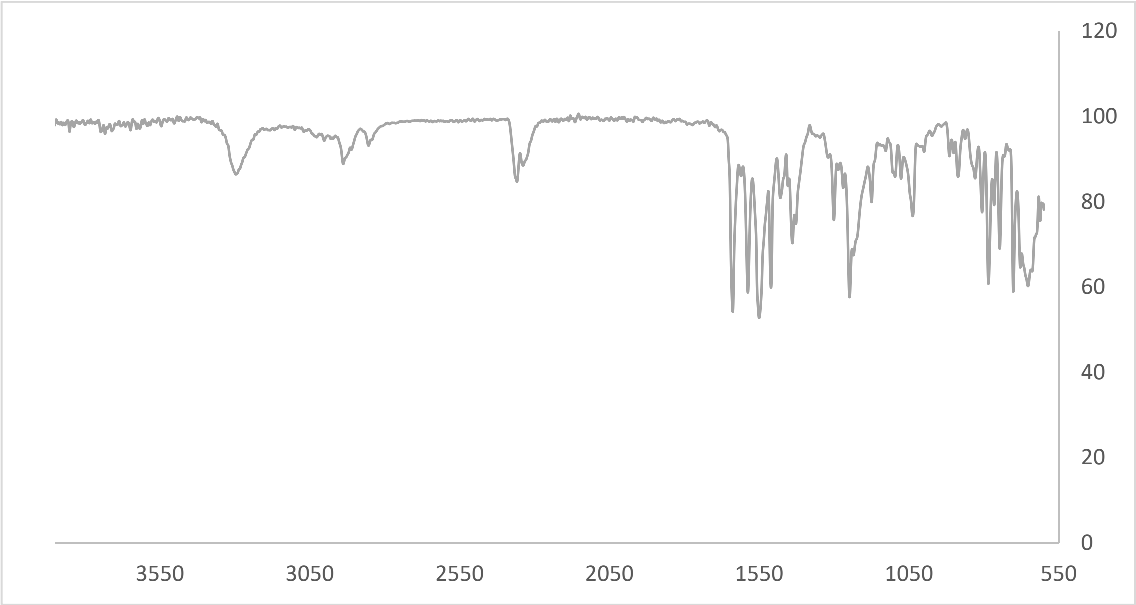
120
100
80
60
40
20
0
3550
3050
2550
2050
1550
1050
550

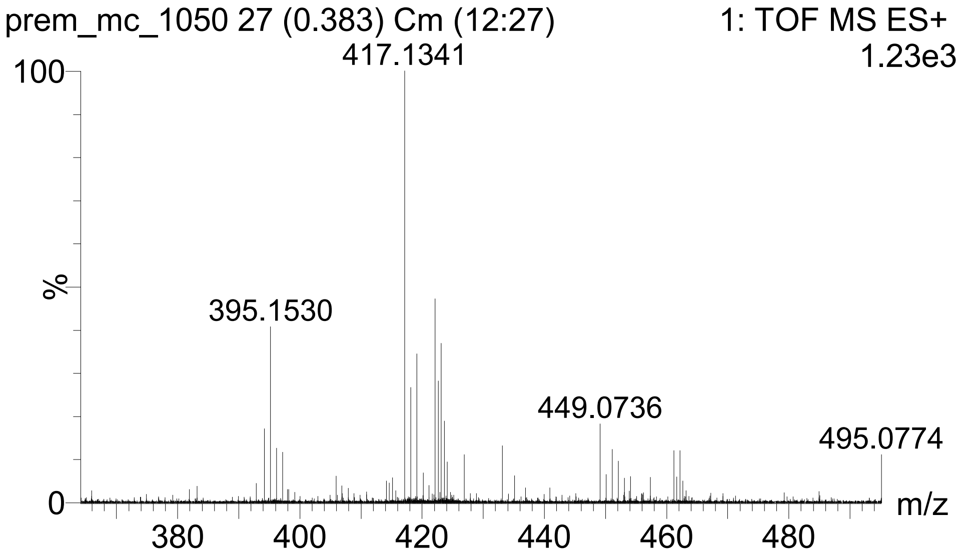
prem_mc_1050 27 (0.383) Cm (12:27)
1: TOF MS ES+
1.23e3
417.1341
100
%
395.1530
449.0736
495.0774
0
m/z
380
400
420
440
460
480

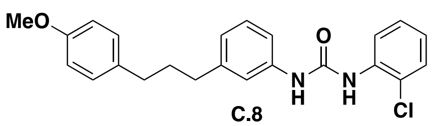
MeO
O
N
H
N
H
Cl

C.8

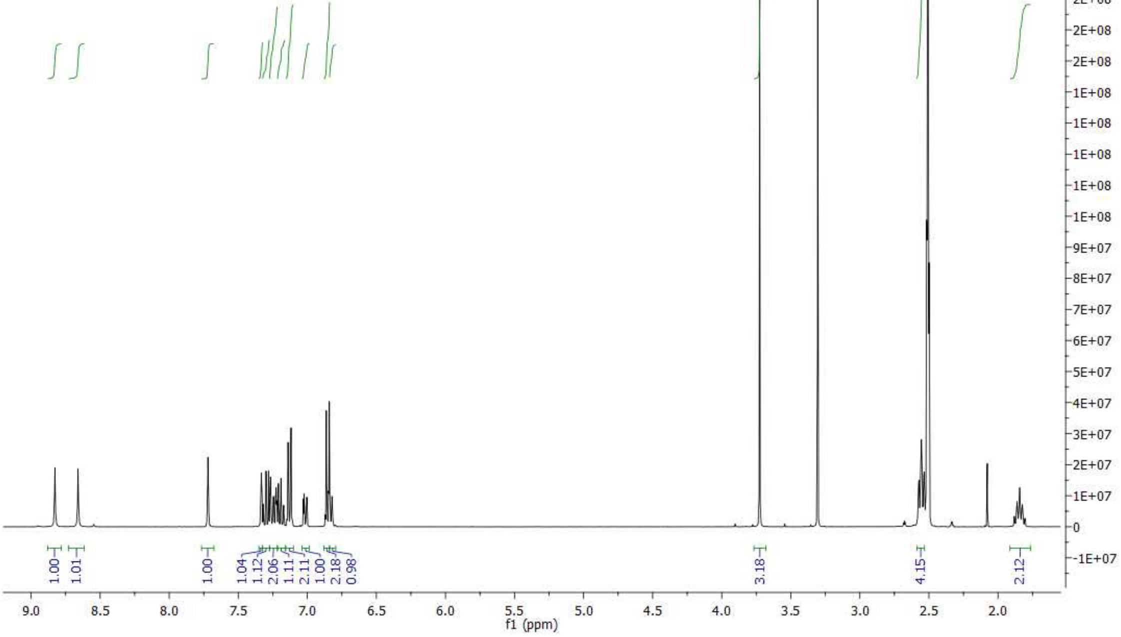
1.00
1.01
1.00
1.04
1.12
2.06
1.11
2.11
1.00
2.18
0.98
3.18
4.15
2.12
f1 (ppm)

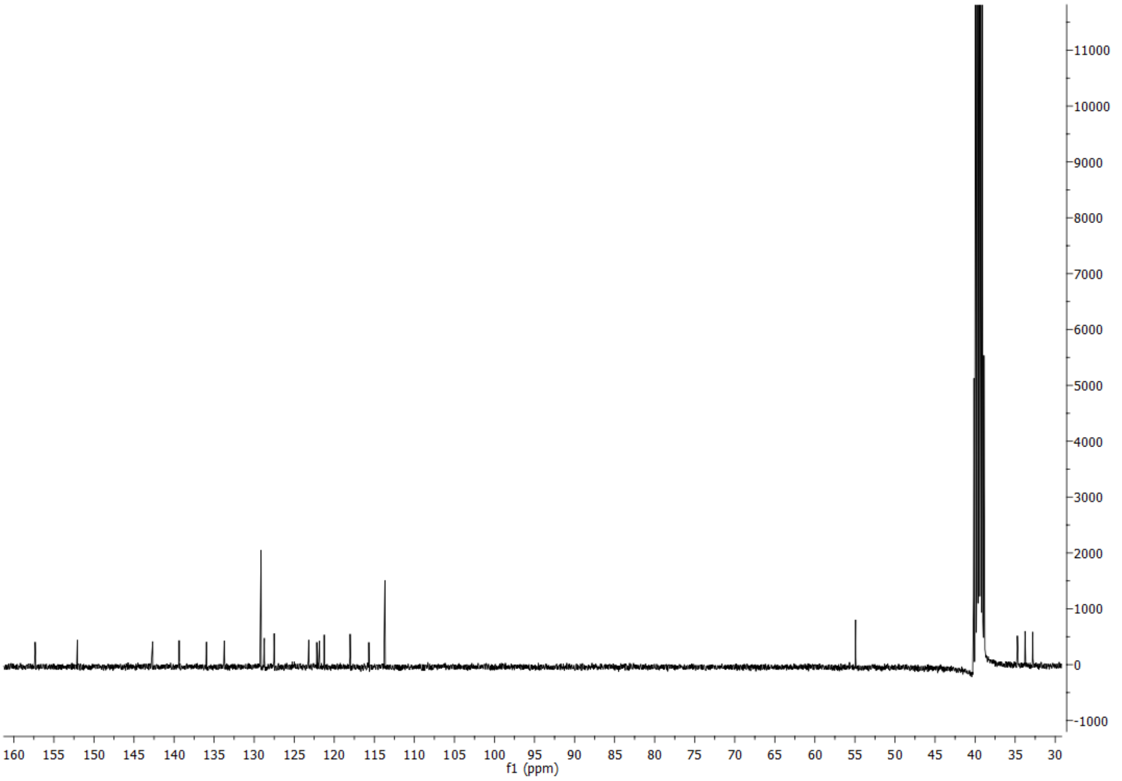
f1 (ppm)

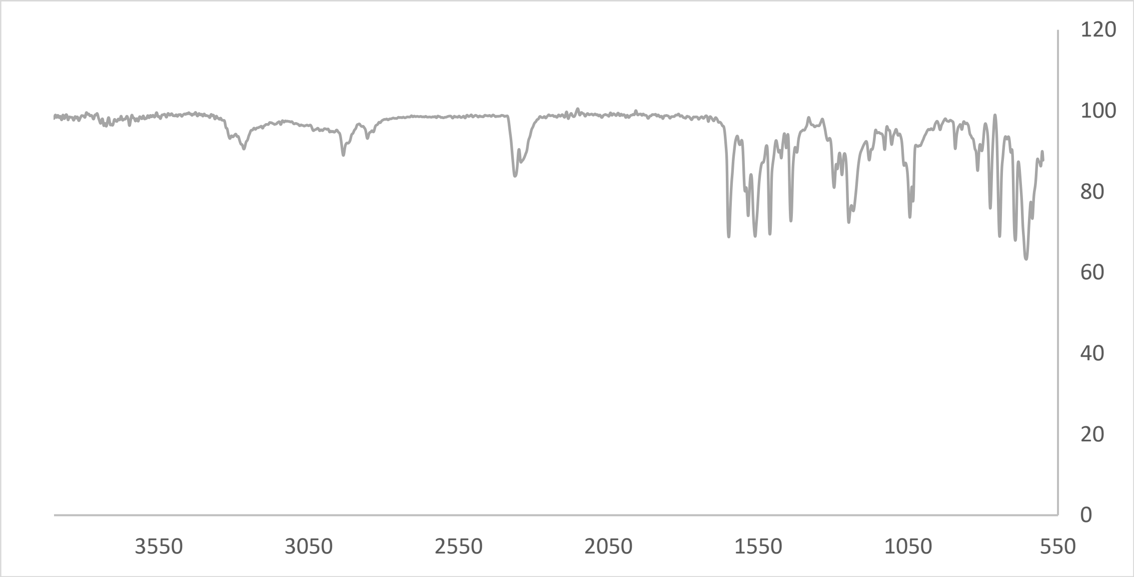
120
100
80
60
40
20
0
3550
3050
2550
2050
1550
1050
550

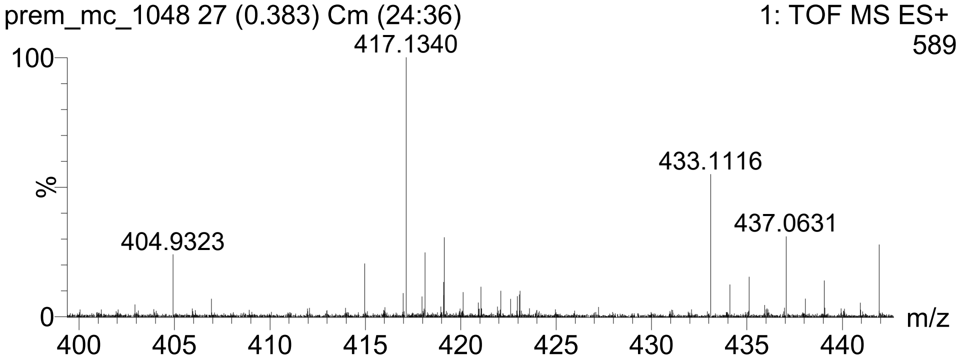
prem_mc_1048 27 (0.383) Cm (24:36)
1: TOF MS ES+
589
100
%
0
417.1340
433.1116
437.0631
404.9323
400
405
410
415
420
425
430
435
440
m/z

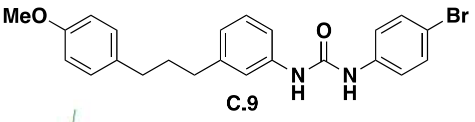
MeO
O
N
H
N
H
Br

C.9

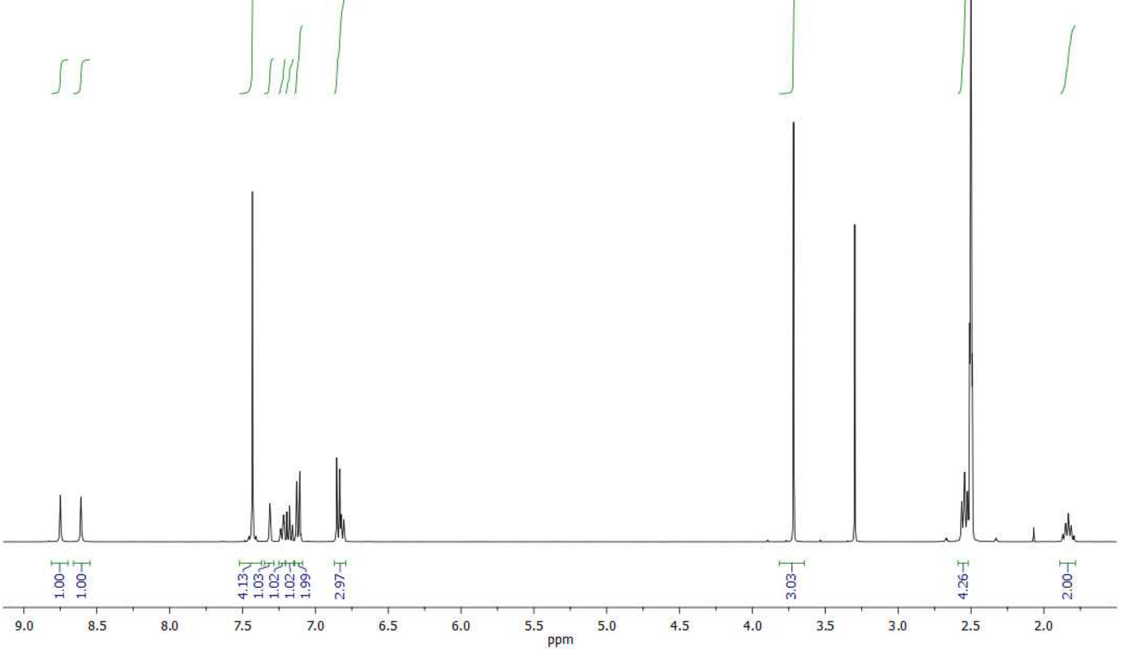
1.00
1.00
4.13
1.03
1.02
1.02
1.99
2.97
3.03
4.26
2.00
9.0
8.5
8.0
7.5
7.0
6.5
6.0
5.5
5.0
4.5
4.0
3.5
3.0
2.5
2.0
ppm

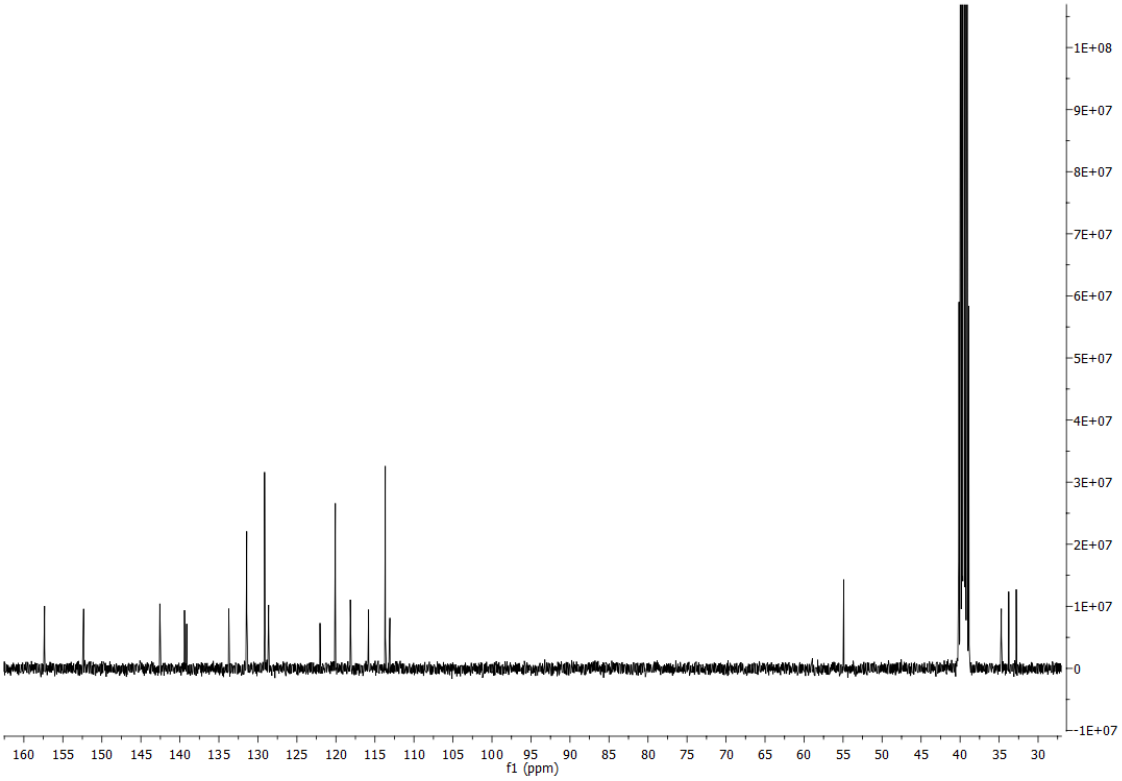
1E+08
9E+07
8E+07
7E+07
6E+07
5E+07
4E+07
3E+07
2E+07
1E+07
0
-1E+07
160 155 150 145 140 135 130 125 120 115 110 105 100 95 90 85 80 75 70 65 60 55 50 45 40 35 30
f1 (ppm)

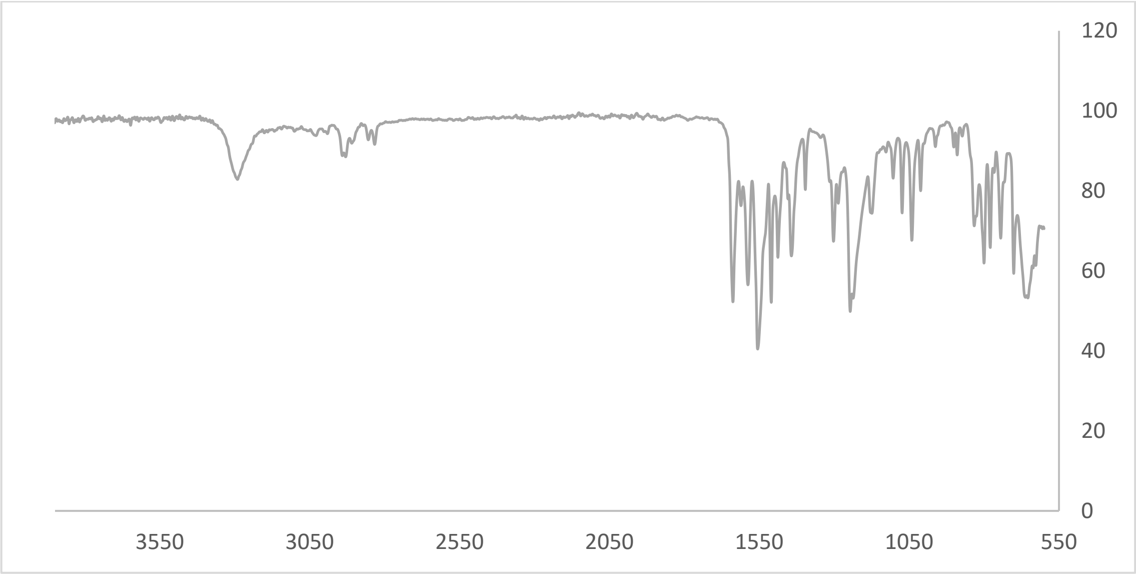
120
100
80
60
40
20
0
3550
3050
2550
2050
1550
1050
550

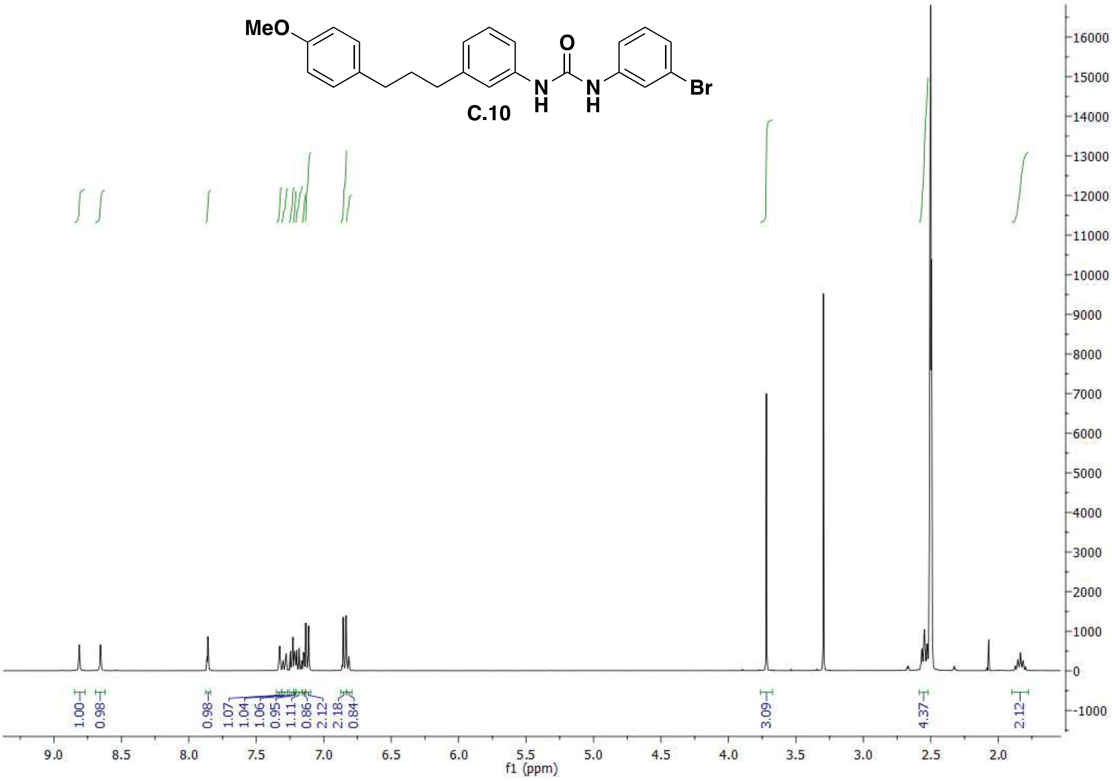
MeO
O
N
H
N
H
Br
C.10
1.00
0.98
0.98
1.07
1.04
1.06
0.95
1.11
0.86
2.12
2.18
0.84
3.09
4.37
2.12
f1 (ppm)

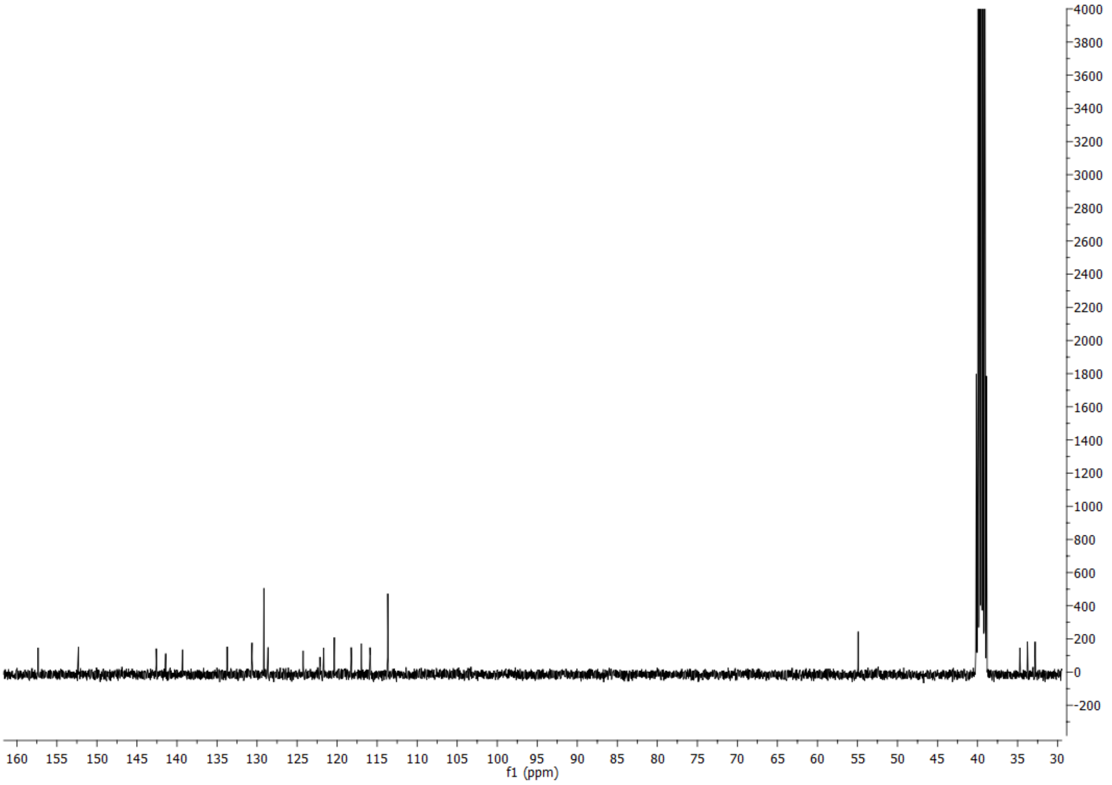
f1 (ppm)

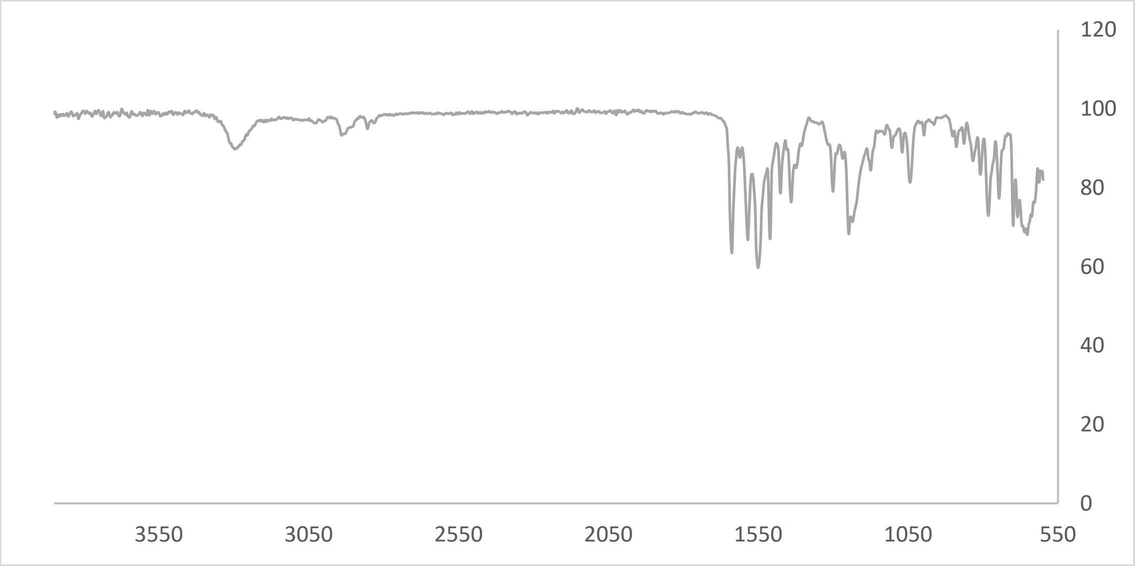
120
100
80
60
40
20
0
3550
3050
2550
2050
1550
1050
550

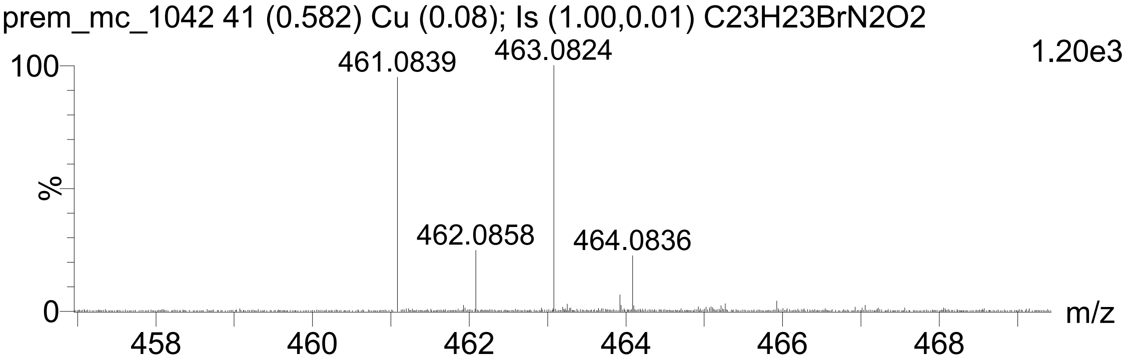
prem_mc_1042 41 (0.582) Cu (0.08); Is (1.00,0.01) C23H23BrN2O2
1.20e3
100
%
0
461.0839
463.0824
462.0858
464.0836
458
460
462
464
466
468
m/z

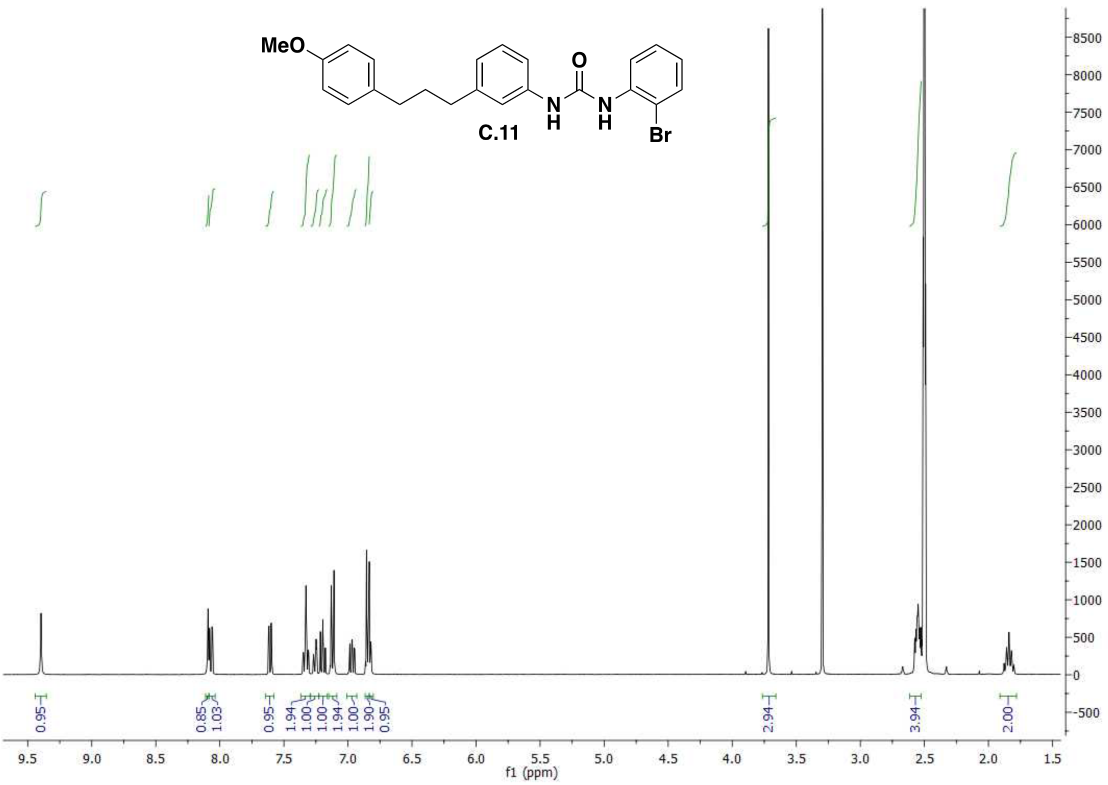
MeO
O
N
H
N
H
Br
C.11
0.95
0.85
1.03
0.95
1.94
1.00
1.00
1.94
1.00
1.90
0.95
2.94
3.94
2.00
f1 (ppm)

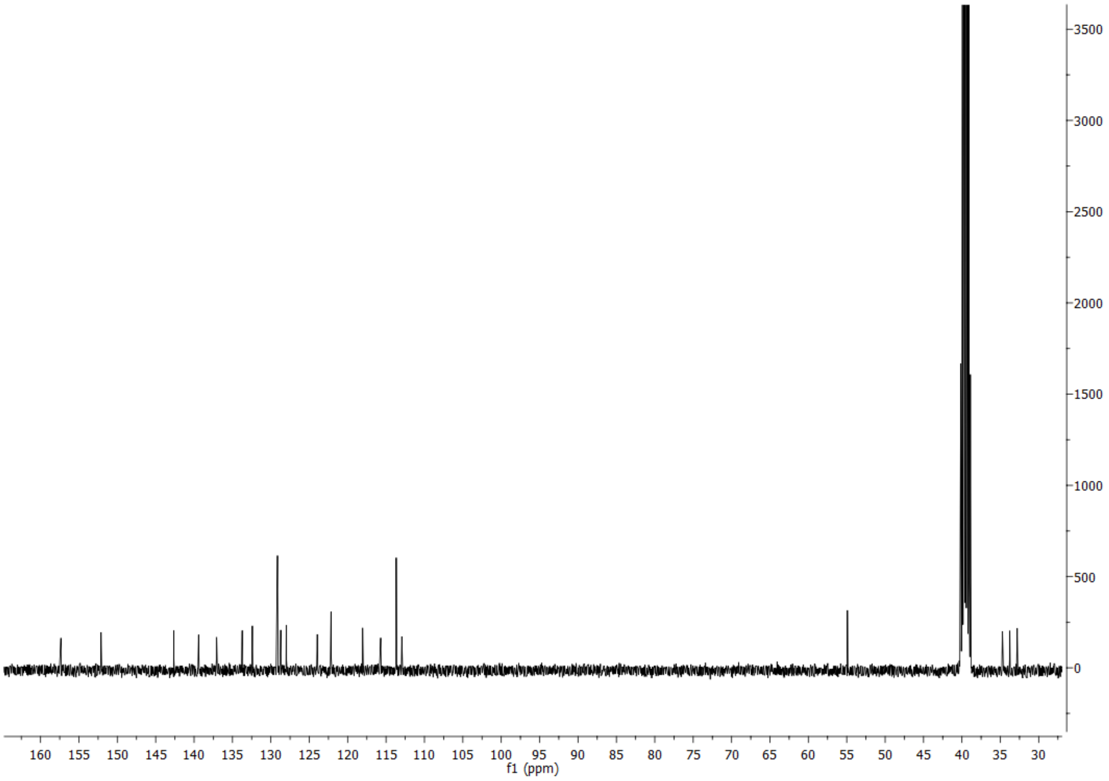
f1 (ppm)

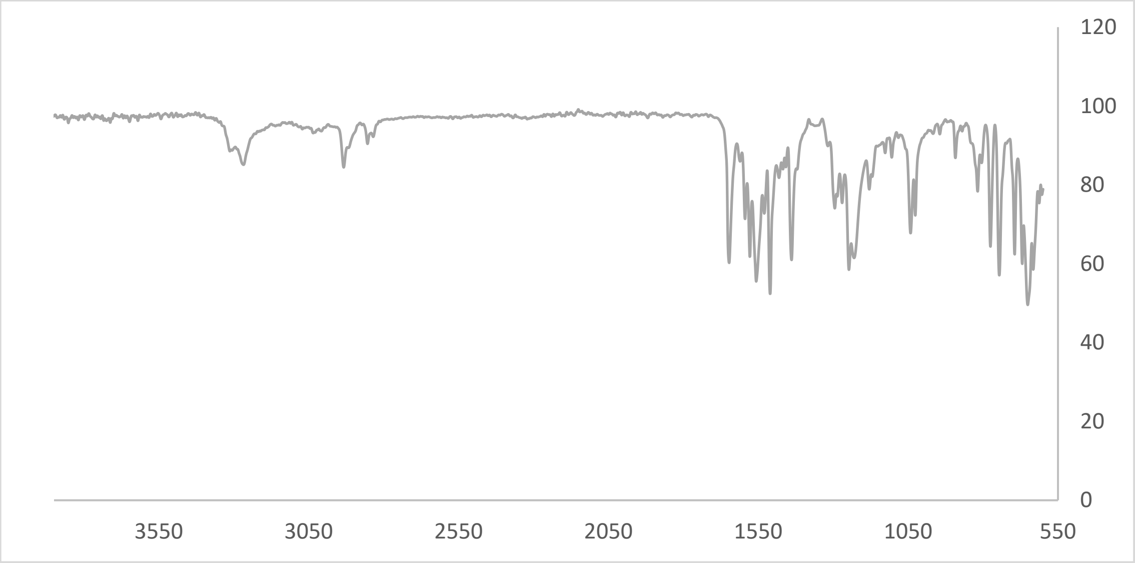
120
100
80
60
40
20
0
3550
3050
2550
2050
1550
1050
550

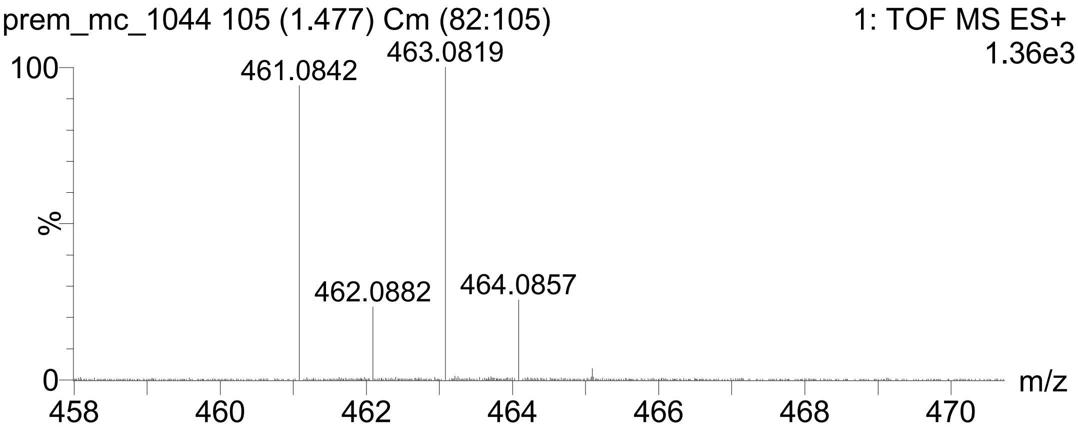
prem_mc_1044 105 (1.477) Cm (82:105)
1: TOF MS ES+
1.36e3
100
%
0
461.0842
463.0819
462.0882
464.0857
m/z
458
460
462
464
466
468
470

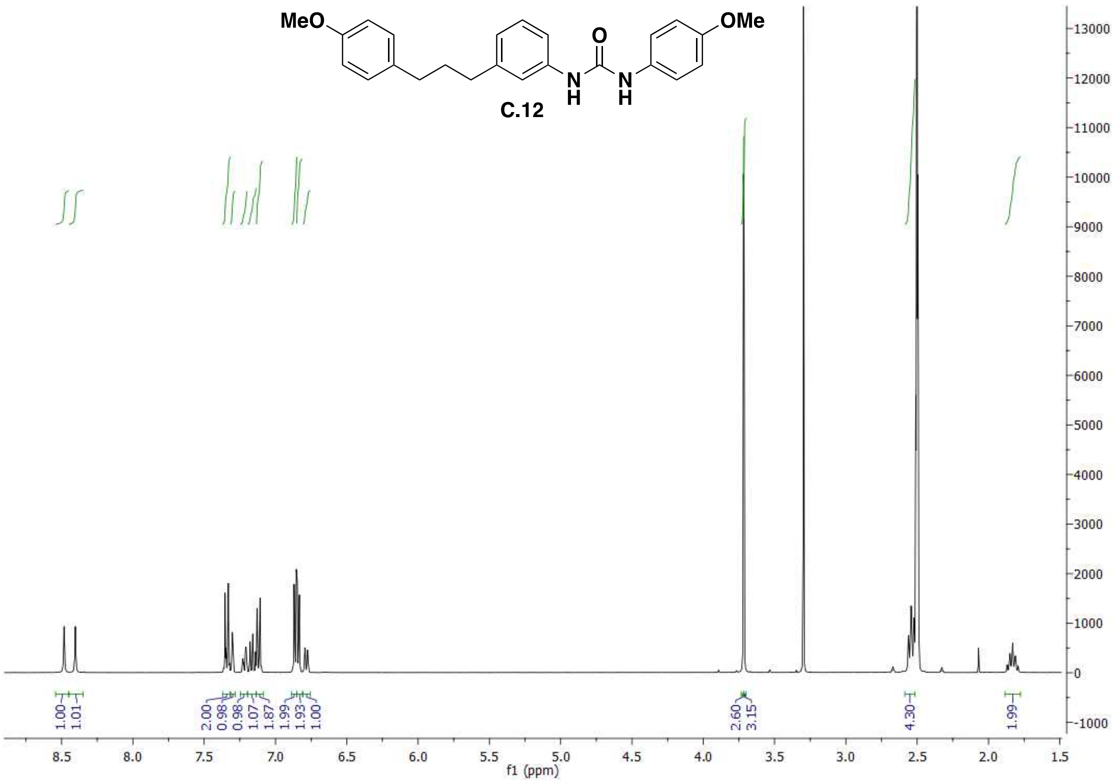
MeO
OMe
O
N
H
N
H
C.12
1.00
1.01
2.00
0.98
0.98
1.07
1.87
1.99
1.93
1.00
2.60
3.15
4.30
1.99
f1 (ppm)

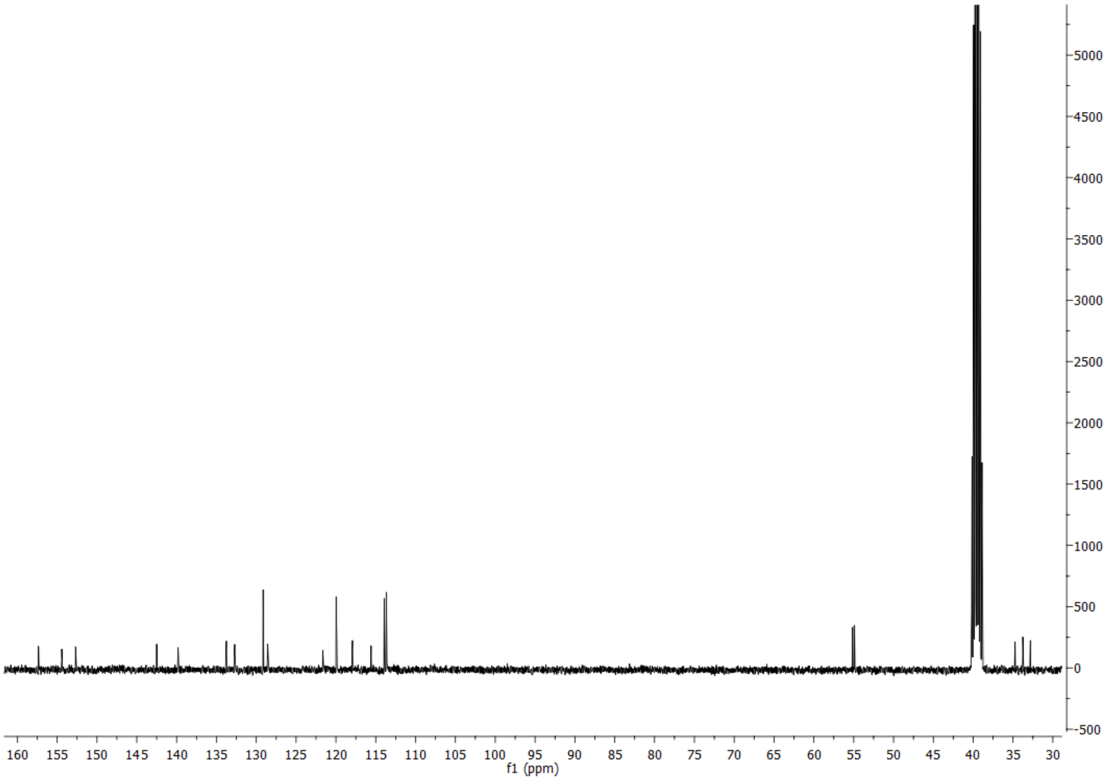
f1 (ppm)

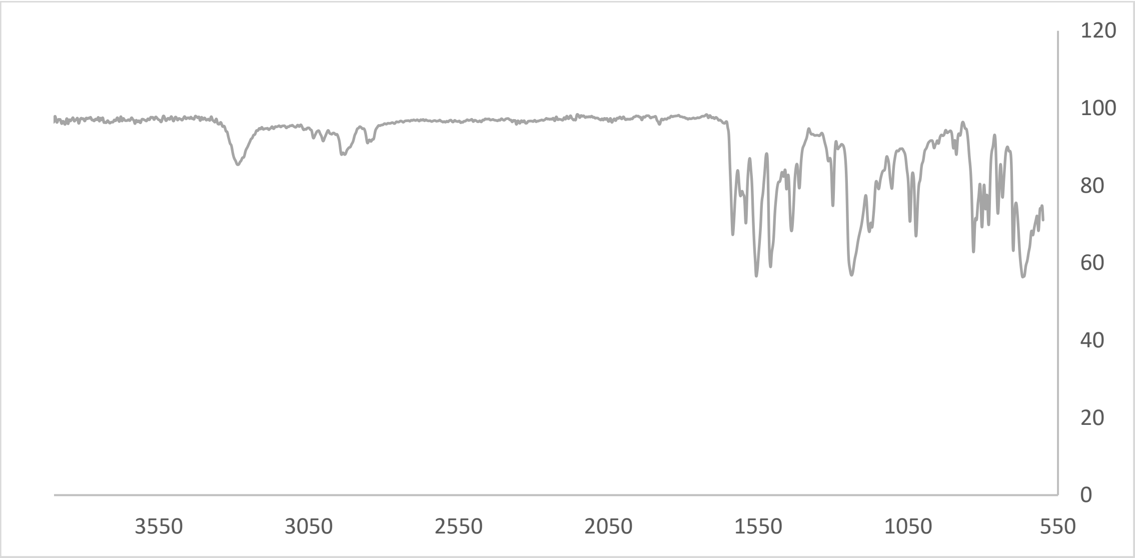
120
100
80
60
40
20
0
3550
3050
2550
2050
1550
1050
550

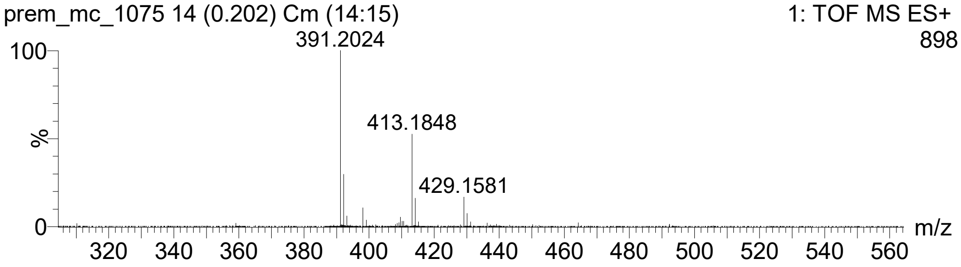
prem_mc_1075 14 (0.202) Cm (14:15)
1: TOF MS ES+
898
391.2024
413.1848
429.1581
100
%
0
m/z
320
340
360
380
400
420
440
460
480
500
520
540
560

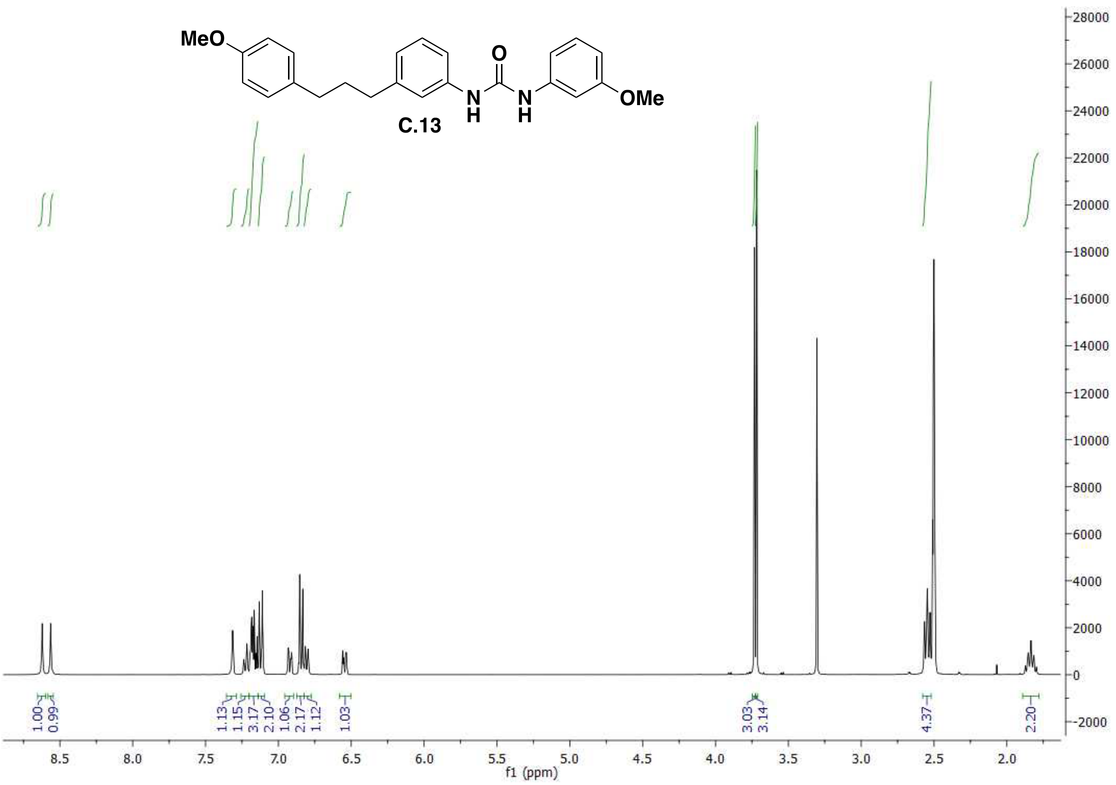
MeO
O
N
H
N
H
OMe
C.13
f1 (ppm)

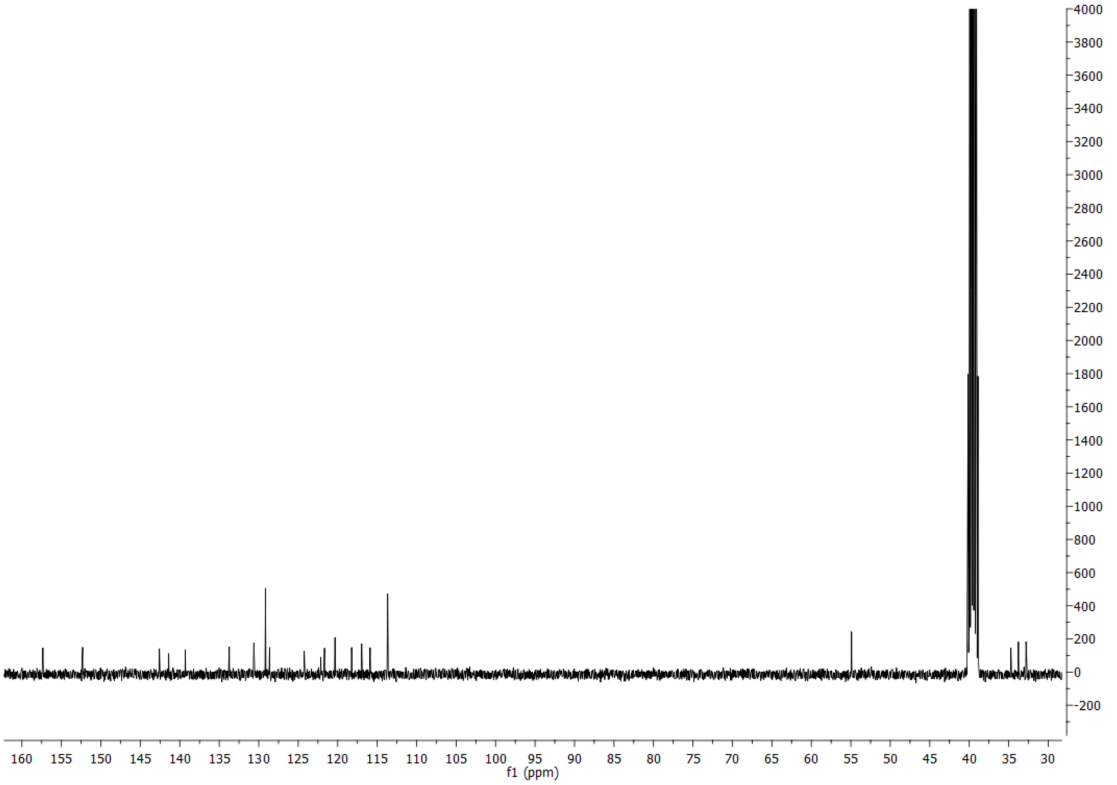
f1 (ppm)

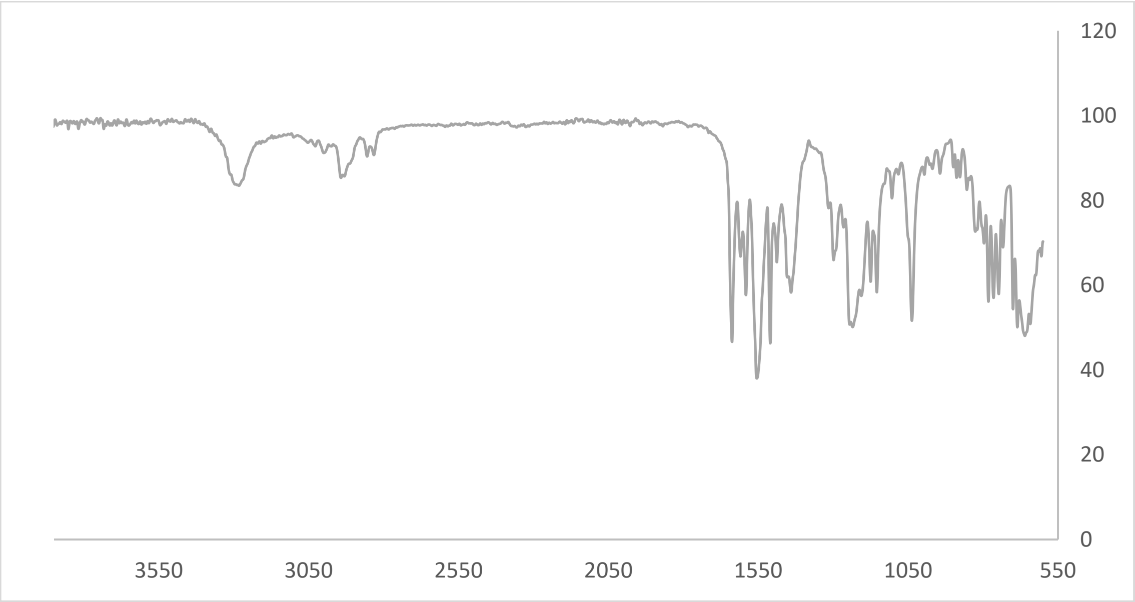
120
100
80
60
40
20
0
3550
3050
2550
2050
1550
1050
550

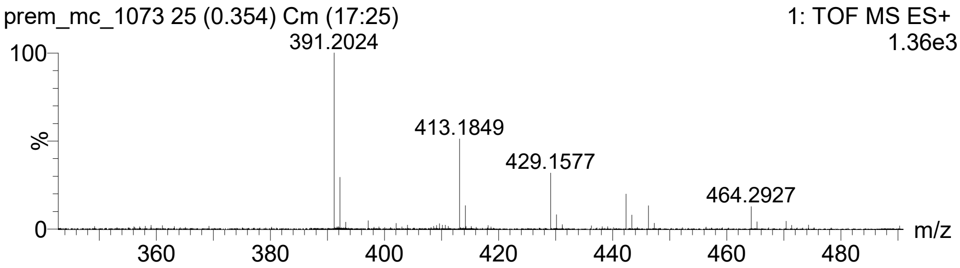
prem_mc_1073 25 (0.354) Cm (17:25)
1: TOF MS ES+
1.36e3
391.2024
413.1849
429.1577
464.2927
100
%
0
360
380
400
420
440
460
480
m/z

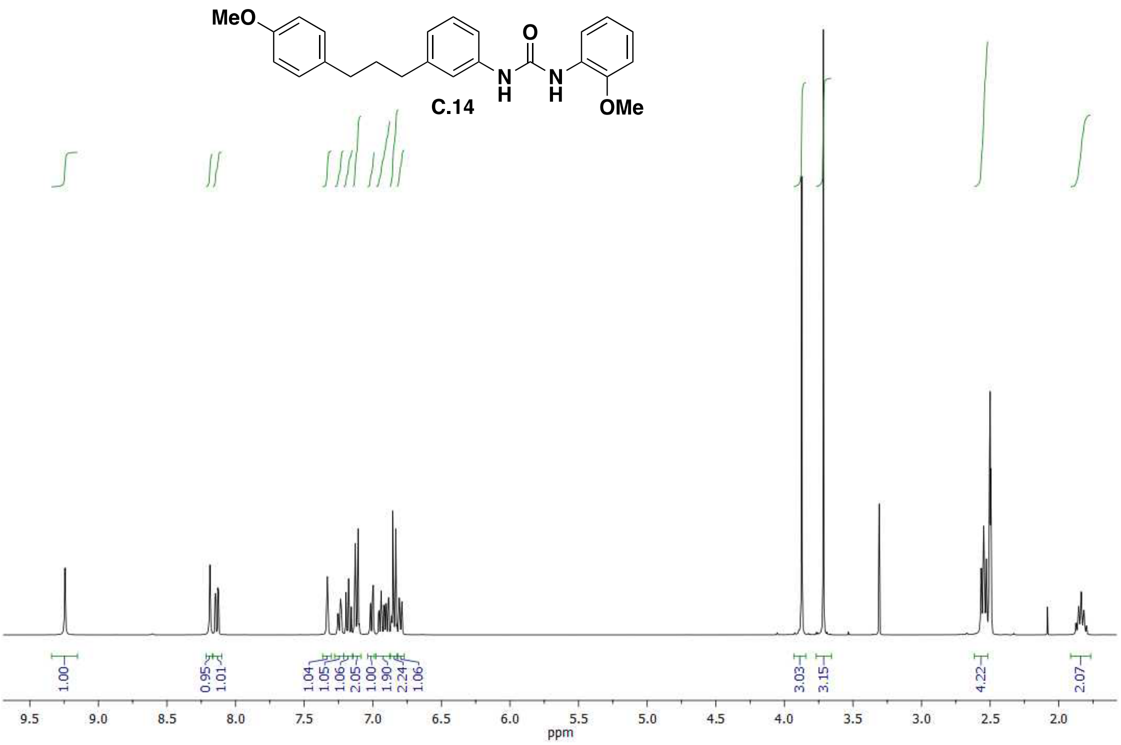
MeO
O
N
H
N
H
OMe
C.14
1.00
0.95
1.01
1.04
1.05
1.06
2.05
1.00
1.90
2.24
1.06
3.03
3.15
4.22
2.07
9.5 9.0 8.5 8.0 7.5 7.0 6.5 6.0 5.5 5.0 4.5 4.0 3.5 3.0 2.5 2.0
ppm

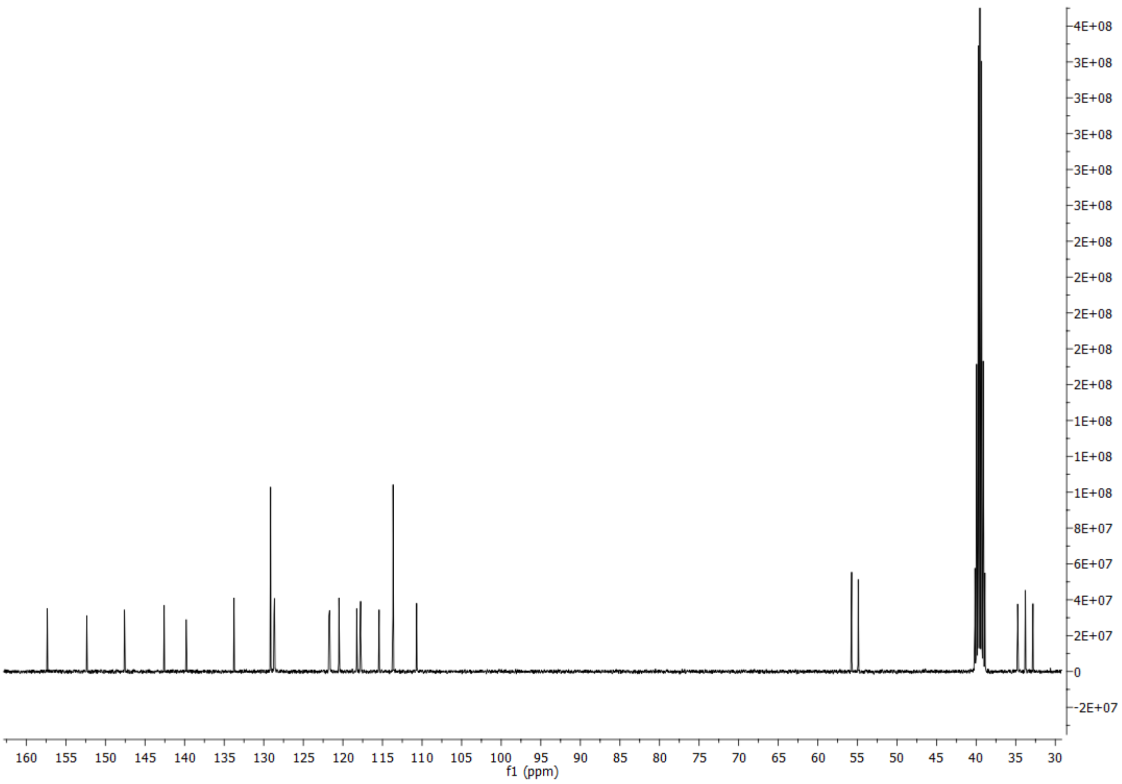
4E+08
3E+08
3E+08
3E+08
3E+08
3E+08
2E+08
2E+08
2E+08
2E+08
2E+08
1E+08
1E+08
1E+08
8E+07
6E+07
4E+07
2E+07
0
-2E+07
160 155 150 145 140 135 130 125 120 115 110 105 100 95 90 85 80 75 70 65 60 55 50 45 40 35 30
f1 (ppm)

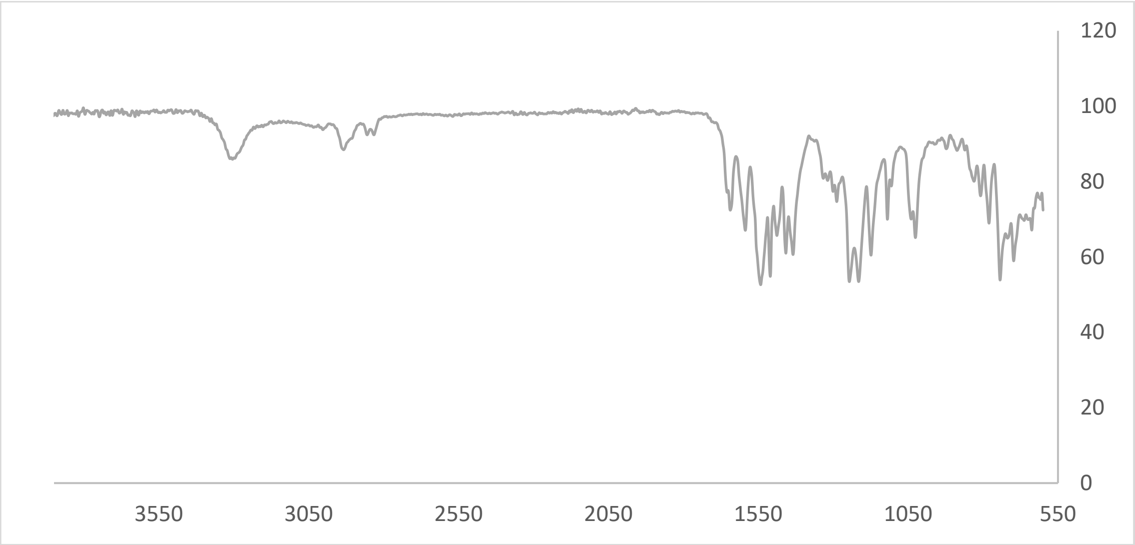
120
100
80
60
40
20
0
3550
3050
2550
2050
1550
1050
550

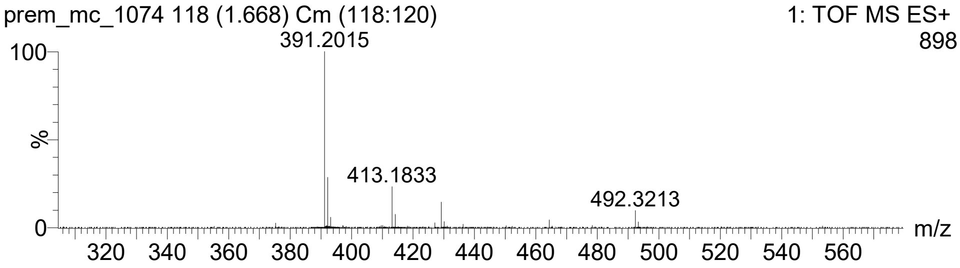
prem_mc_1074 118 (1.668) Cm (118:120)
1: TOF MS ES+
898
391.2015
100
%
413.1833
492.3213
0
m/z
320
340
360
380
400
420
440
460
480
500
520
540
560
